# Raphani Semen Extract Targets the *las* Quorum Sensing System to Suppress *Pseudomonas aeruginosa* Biofilm and Signal Molecule Production

**DOI:** 10.1002/fsn3.72034

**Published:** 2026-08-12

**Authors:** Juntao Xie, Yujing Huang, Honggeng Chen, Zhiyun Xia, Likang Wang, Xin Yu, Jianni Yang, Jun Xu, Hongxia Fan, Junxia Zheng

**Affiliations:** ^1^ School of Biomedical and Pharmaceutical Sciences Guangdong University of Technology Guangzhou People's Republic of China; ^2^ College of Pharmacy Jinan University Guangzhou People's Republic of China; ^3^ Department of Oncology The First Affiliated Hospital of Jinan University Guangzhou People's Republic of China

**Keywords:** *las* system, *Pseudomonas aeruginosa*, quorum sensing, *Raphani Semen*, UPLC‐Q‐Orbitrap‐MS/MS

## Abstract

*Raphanus sativus* L. seeds (Raphani Semen, RS) are traditionally used in Chinese medicine for respiratory and gastrointestinal disorders. *Pseudomonas aeruginosa (P* *. aeruginosa* ) biofilm formation and quorum sensing (QS)‐regulated behaviors contribute significantly to its persistence and resistance, highlighting the need for novel QS‐targeting agents. This study aimed to investigate whether RS extract (RSE) and its characteristic constituents sulforaphane (SFA) and sulforaphene (SFE) inhibit *P. aeruginosa* biofilm and virulence by targeting specific QS pathways. Using sub‐inhibitory concentrations of RSE (≤ 256 μg/mL), SFA and SFE (12.5–100 μM), we evaluated their effects on biofilm formation, bacterial motility, virulence factor production, and signal molecule levels, combined with fluorescent reporter assays, qRT‐PCR, and UPLC‐Q‐Orbitrap‐MS/MS for mechanistic and phytochemical analysis. Results showed that RSE suppressed biofilm formation by 50.93%, impaired motility, and reduced 3‐oxo‐C12‐HSL production. It downregulated key QS genes (*lasR*, *lasA*, *lasB*, *pqsA*, and *pqsR*) and inhibited *lasB*/*pqsA* reporter activity. Phytochemical analysis identified 71 components, and subsequent validation showed that SFA and SFE replicated these effects and exhibited broader QS inhibition across the *las*, *pqs*, and *rhl* systems. SFA (100 μM) demonstrated particularly strong efficacy, suppressing 3‐oxo‐C12‐HSL by 74.29%. Molecular docking indicated that SFA and SFE can bind to LasB, RhlA, and PqsA, albeit with modest affinities. These findings demonstrate that RSE and its active components, SFA and SFE, are potent QS inhibitors that attenuate *P. aeruginosa* pathogenicity primarily by disrupting the *las* system, suggesting their potential as natural anti‐biofilm agents that may target multiple components of the QS system.

## Graphical abstract

Raphani Semen extract (RSE) and its characteristic isothiocyanates sulforaphane (SFA) and sulforaphene (SFE) inhibit *Pseudomonas aeruginosa* biofilm formation and virulence by targeting the las quorum sensing system. RSE reduces the autoinducer 3‐oxo‐C12‐HSL, downregulates QS genes, and suppresses motility and virulence factors. SFA and SFE recapitulate these effects and show synergistic activity with antibiotics. This study positions RSE and its isothiocyanates as natural anti‐virulence agents with potential applications in food safety and infection control.

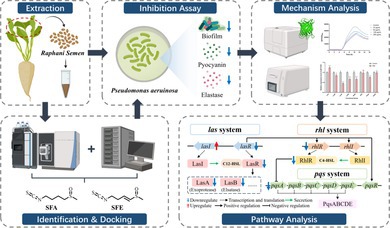

## 1 Introduction

*Raphani Semen* (RS), derived from the desiccated mature seeds of *Raphanus sativus* L. (Cruciferae family), is traditionally known as Lai Fu‐zi in Chinese medical practice. Historically, RS has been used in clinical practice to manage digestive disorders, diarrheal diseases, and respiratory conditions with phlegm congestion (Commission 2020). Modern phytochemical analyses have revealed its rich composition of glucosinolates, isothiocyanates, sinapine derivatives, sinapic acid, flavonoids, polyphenols, amino acids, and fatty acids (Sham et al. 2013; Kim et al. 2014, 2015).

*Pseudomonas aeruginosa* ( *P. aeruginosa* ), a ubiquitous Gram‐negative opportunistic pathogen, poses a significant threat in respiratory infections (Constantino‐Teles et al. 2022; Mesas Vaz et al. 2023). Alarmingly, the World Health Organization (WHO) has classified *P. aeruginosa* as a high priority antibiotic‐resistant pathogen (WHO 2024; Sati et al. 2025). *P. aeruginosa* employs a sophisticated quorum sensing (QS) network to coordinate population‐level behaviors, including biofilm formation and virulence factor secretion, which are critical for its pathogenicity and antibiotic resistance (Papenfort and Bassler 2016; Maiga et al. 2025). Targeting QS with quorum sensing inhibitors (QSIs) has emerged as a promising anti‐Antimicrobial Resistance approach, as it suppresses pathogenicity without exerting selective pressure, thereby minimizing resistance risks (Lu et al. 2022). The *P. aeruginosa* QS system comprises three interconnected pathways: *las*, *rhl*, and *pqs*, with the *las* system (centered on the signal molecule 3‐oxo‐C12‐HSL) acting as the upstream regulator (Vadakkan et al. 2024). The *las* system, centered around the *lasI/lasR* genes and the autoinducer *N*‐(3‐oxododecanoyl)‐L‐homoserine lactone (3‐oxo‐C12‐HSL), initiates the transcriptional cascade that activates downstream virulence genes while simultaneously promoting biofilm matrix production (Mukherjee et al. 2017, 2018; Turkina and Vikström 2018). Targeting the *las* system presents a promising strategy to combat multidrug‐resistant *P. aeruginosa* infections by simultaneously suppressing virulence and biofilm‐associated tolerance.

Natural products represent a rich source of lead compounds with high efficacy and low toxicity, playing a pivotal role in drug discovery and serving as an important reservoir for QSI (Chen et al. 2024; Zhang et al. 2025). In recent years, traditional Chinese medicinal herbs have garnered significant attention in QSI screening efforts due to their favorable safety profiles and eco‐friendly properties (Zeng et al. 2023; Wen et al. 2024). Our preliminary investigations revealed RSE can remarkably suppress *P. aeruginosa* biofilm formation, suggesting its possible QS interference.

Therefore, in this study, the wild type strain of *P. aeruginosa* (PAO1) was used as the model strain to study the inhibitory activity of RSE on its biofilm, virulence factors, and motility ability at sub‐inhibitory concentration. Fluorescence reporter strain and qRT‐PCR technology were further used to elucidate the modulatory effects of RSE on QS systems, and the inhibitory activity of *Raphani Semen* extract on bacterial signal molecule secretion was quantitatively analyzed. UPLC‐Q‐Orbitrap‐MS/MS was used to characterize the chemical components of RSE. This paper provides mechanistic insights into the QS‐inhibitory properties of RS, suggesting its potential as a novel therapeutic strategy against *P. aeruginosa* infections, although further validation using clinical resistant isolates is warranted.

## 2 Materials and Methods

### 2.1 Plant Materials, Extract and Chemical Agents

Raphani Semen was purchased from the Qingping Herbal Medicine Market, Guangzhou, China. A voucher specimen has been deposited at the Laboratory of Natural Medicinal Chemistry of Guangdong University of Technology. The samples purchased for this study were washed with deionized water, and then dried in a ventilated oven at 40°C for 48 h until a constant weight was achieved. The dried seeds were subsequently crushed thickly with a Chinese herbal grinder and passed through a 60 mesh sieve for ultrasonic extraction. Sulforaphane (SFA) was purchased from Shanghai Bide Pharmatech Ltd., and sulforaphene (SFE) was purchased from Chengdu Alfa Biotechnology Co. Ltd. Ethanol, MeOH, MeCN and dimethyl sulfoxide (DMSO) were purchased from Zhiyuan Chemical Reagent Factory. The AHL standards, *N*‐(3‐oxododecanoyl)‐L‐homoserine lactone (3‐oxo‐C12‐HSL), were obtained from Sigma‐Aldrich Chemical Co. (St. Louis, MO, USA). All other chemicals used were molecular biology grade.

### 2.2 Extraction and Isolation

Dried RS (10 g) was accurately weighed and extracted three times with 200 mL of 95% EtOH/H_2_O under ultrasound assistance for 40 min per extraction. The combined filtrate was concentrated under reduced pressure using a rotary evaporator at 45°C to remove the organic solvent. The resulting extract was then freeze‐dried at −80°C to obtain RSE (yield: approximately 0.95 g, 9.5% *w/w* based on dried seeds), which was stored at −20°C.

### 2.3 Strains and Culture Conditions

*P. aeruginosa* strains used in this study included the wild type strain PAO1 (ATCC 15692) and four reporter strains: PAO1 *gfp* (GFP‐labeled wild type strain of *P. aeruginosa* ), PAO1 *lasB*‐*gfp* (containing *lasB*‐*gfp* (ASV) translational reporter fusion), PAO1 *rhlA*‐*gfp* (containing *rhlA*‐*gfp* (ASV) translational reporter fusion), and PAO1 *pqsA*‐*gfp* (containing *pqsA*‐*gfp* (ASV) translational reporter fusion). All strains were kindly provided by Prof. Liang Yang from the Singapore Centre for Environmental Life Sciences Engineering, Nanyang Technological University. For routine cultivation, *P. aeruginosa* PAO1 was grown in 5 mL Luria Bertani (LB) medium, while the reporter strains (PAO1 *gfp*, PAO1 *lasB*‐*gfp*, PAO1 *rhlA*‐*gfp*, and PAO1 *pqsA*‐*gfp*) were cultured in 5 mL LB medium supplemented with 60 μg/mL gentamicin sulphate. All cultures were incubated at 37°C with shaking at 200 rpm for 12 h.

### 2.4 Minimum Inhibitory Concentration (MIC) and Growth Kinetics

The MIC of RSE against *P. aeruginosa* PAO1 was determined using the broth microdilution method following CLSI guidelines. Briefly, overnight cultures of PAO1 were adjusted to an optical density at 600 nm (OD₆₀₀) of 0.1, which corresponds to approximately 1 × 10^8^ CFU/mL, and then diluted 100‐fold in fresh LB broth to obtain a final inoculum of 1 × 10^6^ CFU/mL. Two‐fold serial dilutions of RSE (2, 4, 8, 16, 32, 64, 128, 256, and 512 μg/mL), SFA (6.25, 12.5, 25, 50, and 100 μM), and SFE (62.5, 125, 250, 500, and 1000 μM) were tested. For MIC determination, each 96‐well plate included the following controls: sterile medium alone (media control) to monitor sterility and correct for background absorbance, bacterial suspension without treated (positive control) to confirm normal bacterial growth, and bacterial suspension with the highest concentration of solvent (vehicle control, ≤ 0.5% DMSO) to exclude any solvent‐related growth inhibition. After 24 h incubation at 37°C, the MIC was defined as the lowest concentration exhibiting no visible turbidity. Visible turbidity was observed for RSE up to 512 μg/mL, and for SFA and SFE up to 100 μM. Thus, the MICs were > 512 μg/mL for RSE and > 100 μM for SFA and SFE (Table S2). Azithromycin (positive control) was tested under the same conditions, and its MIC is also provided in Table S2.

**FIGURE 1:**
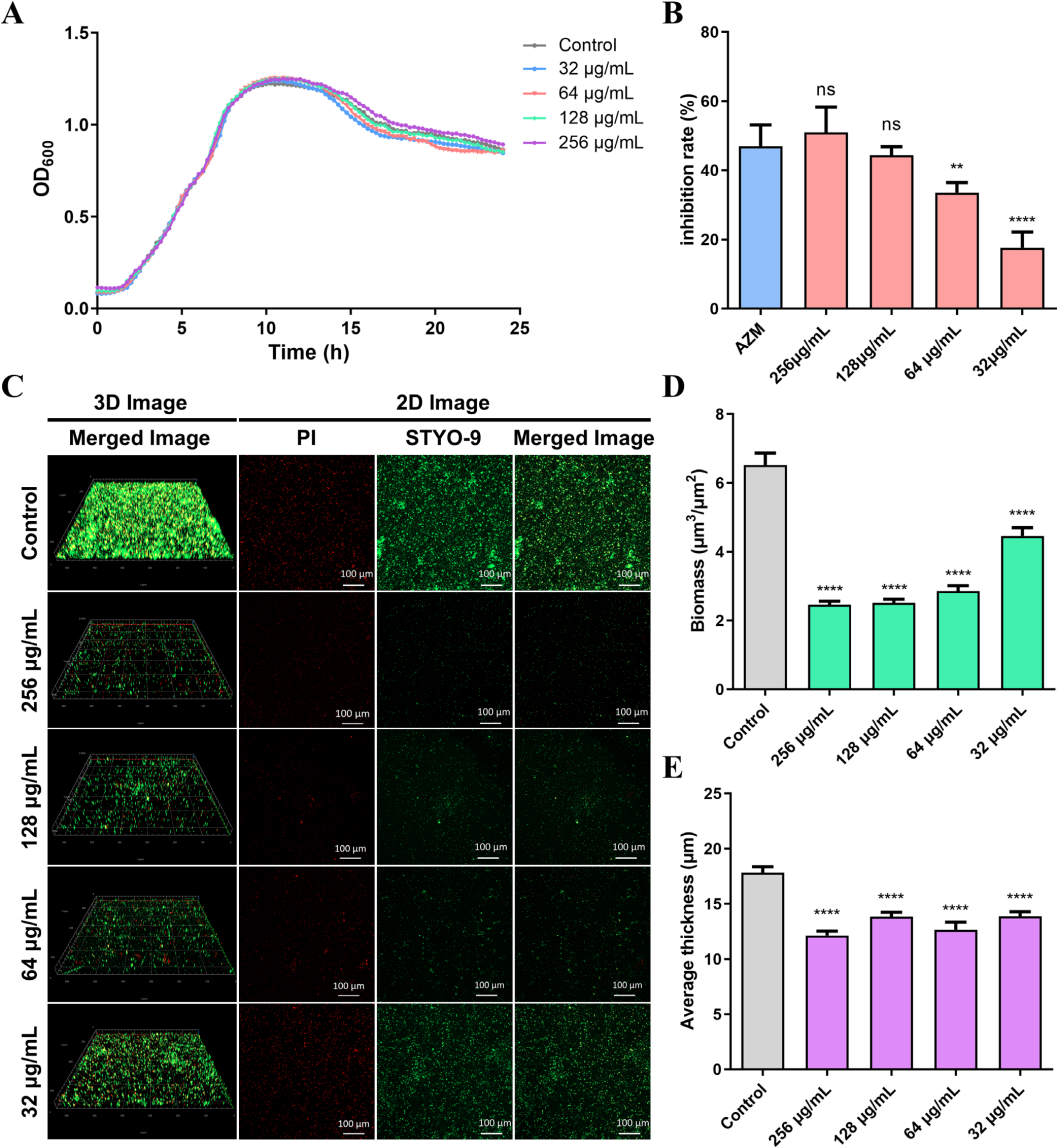
Inhibition of biofilm formation by RSE and CLSM images of PAO1 biofilms. (A) Growth curves of PAO1 treated with RSE at indicated concentrations (32–256 μg/mL). Data are from one representative experiment of three independent biological replicates (each with two technical replicates); no statistical analysis was performed on growth curves. The 512 μg/mL condition was not included in the growth curve experiment but showed no visible turbidity in the MIC plate (MIC > 512 μg/mL; Table S2). (B) Biofilm formation inhibition measured by crystal violet staining. AZM: Azithromycin (positive control, 32 μg/mL). (C) Representative CLSM images of 24 h biofilms from one of three independent experiments. Scale bar = 100 μm. (D, E) COMSTAT analysis of total biofilm biomass (D) and average thickness (E). All quantitative data in (B), (D) and (E) are presented as mean ± SD from three independent biological replicates (*n* = 3). Statistical significance was determined by one‐way ANOVA followed by Tukey's post hoc test. **p < 0.01, ****p < 0.0001 compared to the control.

Growth kinetics were monitored using a Bioscreen C MBR automated growth analyzer (Oy Growth Curves Ab Ltd., Finland): PAO1 cultures (1 × 10^6^ CFU/mL) were treated with sub‐MIC concentrations of RSE (32–256 μg/mL) and absorbance at 600 nm was recorded every 10 min for 24 h at 37°C with continuous shaking. Untreated cultures and medium controls were included. As shown in Figure 1A, no significant growth inhibition was observed at any of these concentrations. Accordingly, RSE concentrations of 32–256 μg/mL were used as sub‐inhibitory doses in subsequent biofilm, motility, and virulence assays. For SFA and SFE, sub‐inhibitory concentrations (6.25–100 μM) were selected based on their MICs (> 1000 μM, Table S2).

### 2.5 Biofilm Formation Inhibition Assay

The effect of RSE, SFA and SFE on PAO1 biofilm formation was evaluated using crystal violet (CV) staining. Overnight PAO1 cultures grown in LB broth at 37°C with shaking at 200 rpm were diluted to OD_600_ = 0.01 in ABTGC minimal medium (AB minimal medium supplemented with Tris–HCl, glucose, and casamino acids), which consisted of AB minimal medium (2 g/L (NH₄)_2_SO₄, 6 g/L Na_2_HPO₄, 3 g/L KH_2_PO₄, 3 g/L NaCl, 1 mM MgCl_2_, 0.1 mM CaCl_2_, 0.01 mM FeCl₃) supplemented with 50 mM Tris–HCl, 0.5% (w/v) glucose, and 0.5% (w/v) casamino acids. Aliquots (100 μL) of this diluted culture were added to 96‐well polystyrene plates containing sub‐MIC concentrations of RSE (32–256 μg/mL), SFA (6.25–100 μM), or SFE (6.25–100 μM). Following 24 h static incubation at 37°C, planktonic cells were aspirated, and adherent biofilms were washed twice with PBS, fixed with 200 μL methanol (15 min), and stained with 0.1% (w/v) CV solution (200 μL/well, 15 min). Excess dye was removed by gentle rinsing with deionized water, and bound CV was solubilized with 33% (*v/v*) acetic acid. Absorbance at 570 nm was measured using a microplate reader (Varioskan LUX, Thermo Fisher Scientific). Biofilm inhibition (%) was calculated relative to untreated controls using the formula:

$$\text{Inhibition}\;\left(\%\right)=\left(1-\frac{{\mathrm{OD}}_{570,\text{treated}}}{{\mathrm{OD}}_{570,\text{control}}}\right)\times 100$$

Experiments were performed in triplicate with technical replicates (*n* = 3).

### 2.6 Microscopic Analysis

The effect of RSE on PAO1 biofilm formation was observed using confocal laser scanning microscopy (CLSM). Briefly, the overnight culture of *P. aeruginosa* PAO1 was adjusted to an OD_600_ of 0.01. LB agar containing RSE (32–256 μg/mL) was dispensed into 20 mm‐diameter Petri dishes (20 mL per dish). After the agar solidified, 1 μL of bacterial culture (diluted to approximately 1 × 10^6^ CFU/mL) was spotted onto the surface. The plates were incubated at 37°C for 24 h. Following a 24 h incubation at 37°C, the planktonic bacteria and culture medium were carefully removed. The adherent biofilm was then gently rinsed twice with sterile phosphate‐buffered saline (PBS) to remove non‐adherent cells. For viability staining, a SYTO 9/PI dye mixture was prepared using the LIVE/DEAD BacLight Bacterial Viability Kit (Thermo Fisher Scientific) according to the manufacturer's instructions. Subsequently, 300 μL of the dye mixture was added to each Petri dish to cover the biofilm and incubated in the dark for 15 min. Finally, the stain was aspirated, and the biofilm was washed three times with PBS to remove any residual dye prior to imaging. Biofilms formed were visualized via CLSM, and COMSTAT2 software was used to quantify biofilm biomass and average thickness.

### 2.7 Swimming and Swarming Motility

Swimming and swarming motility assays were performed as described by Packiavathy et al. (2012). For the swimming assay, overnight culture of *P. aeruginosa* PAO1 were spot‐inoculated at the center of swimming agar plates, which consisted of LB broth supplemented with 0.3% agar and 20% filter‐sterilized glucose, and contained various sub‐inhibitory concentrations of RSE (32, 64, and 128 μg/mL). For the swarming assays, overnight cultures were similarly spot‐inoculated at the center of the swarming agar plates, with the same composition as swimming agar (LB broth, 0.3% agar, and 20% filter‐sterilized glucose) and supplemented with the above concentrations of RSE. All plates were incubated at 37°C for 24 h, after which the diameters of the swimming and swarming zones were measured and recorded.

### 2.8 Influence of RSE on Virulence Factor Production

#### 2.8.1 Elastase Activity

Overnight PAO1 cultures were adjusted to an OD_600_ of 0.01 in ABTGC medium. RSE was then added, and the cultures were incubated at 37°C with shaking conditions (200 rpm) for 24 h. A 0.8 mL aliquot of culture supernatant was sampled and centrifuged at 10,000 rpm for 10 min. Elastase activity was determined using an EnzChek elastase assay kit (Invitrogen, USA). Fluorescence signals (excitation: 400 nm; emission: 450 nm) were recorded every 10 min for 4 h using a Tecan Infinite 200 Pro plate reader.

#### 2.8.2 Pyocyanin Assay

An 8 mL aliquot of supernatant was transferred to a centrifuge tube, and 2 mL chloroform was added. After vigorous vortexing for 5 min, the mixture was incubated at room temperature for 10 min, then centrifuged at 4000 × g for 10 min at 4°C. The chloroform phase was carefully transferred to new tubes and mixed with 0.3 volumes of 0.2 M dilute hydrochloric acid. Following thorough mixing, the upper acidic aqueous phase was collected, and its absorbance was measured at 520 nm.

#### 2.8.3 Rhamnolipid Assay

Aliquots (2 mL) of culture supernatant were mixed with 4 mL diethyl ether (2:1 *v/v*) in centrifuge tubes. After vigorous vortexing for 5 min, samples were incubated at room temperature for 10 min, then centrifuged at 4000 × g for 10 min at 4°C. The ether phase was collected, and the extraction was repeated twice. Combined ether extracts were evaporated to dryness under a stream of nitrogen, and the residue was reconstituted in 500 μL ultrapure water by heating at 80°C and vortexing. For colorimetric analysis, 100 μL aliquots were reacted with 900 μL orcinol reagent (0.19% w/v in 50% sulfuric acid) and incubated at 80°C for 30 min. After cooling to room temperature, absorbance was measured at 421 nm, and results were normalized to the final OD_600_ values of bacterial cultures.

#### 2.8.4 Extracellular Polysaccharide Assay

The inhibitory effect of RSE on the extracellular polysaccharide secretion of PAO1 was determined using the phenol‐sulphate acid method. Briefly, 5 mL culture supernatant was transferred to a centrifuge tube, and 15 mL of anhydrous ethanol was added. The mixture was placed in a 4°C refrigerator for 24 h to allow precipitation. Extracellular polysaccharides were collected by centrifugation at 12,000 rpm for 10 min. The precipitation was redissolved in 5 mL of ultrapure water, and a 1 mL aliquot of this solution was transferred to a new tube. After adding 0.5 mL 5% phenol solution, 2.5 mL concentrated sulfuric acid was immediately added. The mixture was vortexed thoroughly and heated in a 90°C water bath for 30 min. After cooling, the absorbance was measured at 490 nm.

### 2.9 UPLC‐Q‐Orbitrap‐MS/MS Analysis of AHL

*N*‐acyl homoserine lactones (AHLs) were extracted from culture supernatants of *P. aeruginosa* PAO1 incubated with or without RSE. Briefly, culture supernatants were centrifuged at 12,000 × *g* for 10 min at 4°C, followed by three rounds of liquid–liquid extraction with acidified ethyl acetate (acidified with 0.1% formic acid, 1:1 *v/v* ratio). The combined organic phases were concentrated under reduced pressure and reconstituted in acidified methanol (0.1% formic acid). The identity and quantification of 3‐oxo‐C_12_‐HSL were performed using a Thermo Scientific Hypersil GOLD C18 column (100 × 2.1 mm, 1.9 μm) on a UPLC‐Q‐Orbitrap‐MS/MS (Thermo Fisher, USA). The mobile phase consisted of water with 0.1% formic acid (buffer A) and acetonitrile (buffer B) at a flow rate of 0.25 mL/min. A 2 μL aliquot of each sample was injected, and separation was achieved via gradient elution: 0.0–5.0 min, 5%–15% B; 5.0–15.0 min, 15%–30% B; 15.0–25.0 min, 30%–100% B, with column temperature maintained at 40°C. Mass spectrometry was conducted using an electrospray ionization (ESI) source operating in both positive and negative ion modes over a mass range of *m/z* 70–1000. Full‐scan MS data were acquired at a resolution of 70,000 (FWHM at *m/z* 200), and targeted MS/MS fragmentation was performed for structural confirmation. Quantification of 3‐oxo‐C_12_‐HSL was achieved using external calibration curves generated from authentic standards, with peak areas normalized to retention times and mass spectral data.

### 2.10 Expression Assay of Quorum Sensing (QS)‐Related Genes Using qRT‐PCR

QS‐regulated genes, including *lasI*, *lasR*, *lasA*, *lasB*, *rhlR*, *rhlI*, *pqsA*, and *pq*sR, were identified from GenBank. Specific qRT‐PCR primers (Table S1) were designed using Primer Premier 5.0 software. The specificity of the qRT‐PCR primers was confirmed by agarose gel electrophoresis of the PCR products (Figure S4). The amplification efficiency and specificity were further verified by the primary curves (Figure S5) and melting curve analysis (Figure S6), which showed single peaks for all target genes. PAO1 cultures were grown in LB medium supplemented with or without sub‐MIC RSE at 37°C with shaking (200 rpm) for 24 h. Total RNA was extracted using the RNeasy Protect Bacteria Mini Kit (Qiagen) according to the manufacturer's protocol. For genomic DNA elimination, 1 μg RNA was treated with gDNA Eraser (PrimeScript RT Master Mix, TaKaRa) at 42°C for 2 min, followed by reverse transcription into cDNA using the same kit at 37°C for 15 min. qRT‐PCR was performed on a LightCycler 480 II system (Roche) with TB Green Premix Ex Taq (TaKaRa), using the following cycling conditions: 95°C for 30 s, followed by 40 cycles of 95°C for 5 s and 60°C for 30 s. Gene expression levels were normalized to the housekeeping gene 16S rRNA and calculated using the 2^−ΔΔCt^ method.

### 2.11 Chemical Composition Analysis

Chemical profiling of *Raphani Semen* extracts was performed using a UPLC‐ Q‐Exactive Orbitrap‐MS/MS (Thermo Fisher, CA, USA) equipped with a heated electrospray ionization (HESI) probe. Chromatographic separation was achieved on a Thermo Scientific Hypersil GOLD C18 (100 × 2.1 mm, 1.9 μm) operated in both negative and positive ionization modes. The mobile phase consisted of a combination of 0.1% formic acid in water (buffer A) and acetonitrile (buffer B), with a flow rate of 250 μL/min. A 2 μL aliquot of each sample was injected, and gradient elution was programmed as follows: 0–25 min (5%–100% B), 25–26 min (100% B), 26–30 min (100%–5% B for re‐equilibration). The column oven and autosampler tray were maintained at 45°C and 10°C, respectively. The data were acquired in both negative and positive ionization modes with dependent MS/MS acquisition at ranges of m/z 70–1000, respectively. The full scan and fragment spectra were collected at a resolution of 70,000 and 17,500, respectively. Using the information for retention time and characteristic product ions, MS1 and MS2 mass errors of > 5 ppm were excluded.

### 2.12 Molecular Docking

Molecular docking studies were performed using the Schrödinger Suite 2023‐4 to predict the binding modes and affinities of SFA and SFE with QS‐related target proteins. The crystal structures of LasB (Elastase, PDB ID: 8CC4), RhlA (Rhamnosyltransferase 1, PDB ID: 8IK2), and PqsA (Anthranilate‐CoA ligase, PDB ID: 5OE5) were retrieved from the RCSB Protein Data Bank (PDB). Prior to docking, protein structures were optimized using the Protein Preparation Wizard. This process included the removal of crystallographic water molecules, the addition of hydrogen atoms, and energy minimization using the OPLS4 force field to optimize the hydrogen bonding network. The 3D structures of the ligands, sulforaphane (SFA) and sulforaphene (SFE), were prepared using LigPrep to generate biologically relevant ionization states and low‐energy 3D geometries. Receptor grids were generated using the Glide module. The grid box size was set to 20 Å × 20 Å × 20 Å, centered on the geometric centroid of the co‐crystallized ligand for LasB and PqsA. For RhlA, which lacks a co‐crystallized ligand, the grid was centered on the conserved active site residues. The exact grid coordinates were determined automatically by the software based on the selected binding site. Molecular docking was subsequently conducted using the Glide module in Standard Precision (SP) mode with default parameters. The binding affinity was evaluated based on the docking score (kcal/mol), and the final binding modes and molecular interactions were visualized using PyMOL.

### 2.13 Statistical Analysis

All experiments were performed with at least three independent biological replicates (*n* = 3), unless otherwise stated in the figure legends. For each biological replicate, two to three technical replicates were included, and the mean of technical replicates was used as one data point. Data are presented as mean ± standard deviation (SD). For comparisons between two groups, an unpaired two‐tailed Student's *t*‐test was applied. For comparisons among three or more groups, one‐way analysis of variance (ANOVA) was used, followed by Tukey's post hoc test for multiple comparisons against the control group. A *p*‐value < 0.05 was considered statistically significant. Significance levels are indicated as *p < 0.05, **p < 0.01, ***p < 0.001, and ****p < 0.0001. All statistical analyses were carried out using GraphPad Prism10.1.2 (GraphPad Software, San Diego, CA, USA).

## 3 Results

### 3.1 MIC and Biofilm Formation Inhibition of RSE

To evaluate the effect of RSE on bacterial growth, the MIC against *P. aeruginosa* PAO1 was determined using broth microdilution. Results showed that RSE did not exhibit significant growth‐inhibitory activity at concentrations up to 512 μg/mL, with visible turbidity at the highest concentration tested (512 μg/mL). Thus, the MIC of RSE against PAO1 was determined to be > 512 μg/mL (Table S2). Growth curve analysis (Figure 1A) further confirmed that RSE at concentrations up to 256 μg/mL (those used in subsequent assays) did not significantly affect bacterial growth, with no statistical differences between treated and untreated cultures. Of note, since no growth inhibition was observed at any concentration tested, these compounds exhibited neither bactericidal nor bacteriostatic activity against PAO1 under the experimental conditions. Based on these findings, sub‐inhibitory concentrations (≤ 256 μg/mL) were selected for subsequent experiments to exclude potential interference from direct bactericidal or bacteriostatic effects, focusing specifically on anti‐biofilm activity.

The effect of RSE on biofilm formation was assessed using crystal violet staining. At sub‐inhibitory concentrations, RSE exhibited a dose‐dependent inhibitory effect on PAO1 biofilm formation (Figure 1B). Among the tested concentrations, 256 μg/mL RSE showed the most prominent activity, reducing biofilm biomass by approximately 50% relative to untreated controls.

Confocal laser scanning microscopy (CLSM) was used to visually and quantitatively validate the anti‐biofilm activity of RSE. Representative CLSM images of 24 h biofilms (Figure 1C) showed marked reductions in the total bacterial population (green‐fluorescent SYTO 9 staining) and in adherent cell density in RSE‐treated groups compared to controls. No proportional increase in red‐fluorescent (PI‐positive) cells was observed, indicating that the reduction in biofilm biomass resulted primarily from impaired bacterial attachment and biofilm formation rather than from direct bactericidal effects. This conclusion is consistent with the MIC and growth curve data, which showed no bactericidal activity. Quantitative analysis using COMSTAT2 software further confirmed these observations, revealing significant decreases in total biofilm biomass (Figure 1D) and average thickness (Figure 1E) in RSE‐treated samples. Statistical analysis by *t*‐test indicated that these reductions were significant at varying levels (*p* < 0.05 to *p* < 0.0001) compared to controls. Together, these results demonstrate that RSE inhibits PAO1 biofilm formation in a dose‐dependent manner, and this activity is independent of direct growth‐inhibitory effects.

### 3.2 Influence of RSE on Swimming and Swarming Motility

Bacterial motility is a critical virulence determinant in *P. aeruginosa* , facilitating initial surface attachment, biofilm formation, and tissue invasion (Liao et al. 2022). Specifically, flagellum‐driven swimming motility enables planktonic cells to navigate liquid environments and colonize abiotic/biotic surfaces, while QS‐regulated swarming motility promotes collective migration across solid substrates via coordinated flagellar movement (Curran et al. 2017). Given the observed anti‐biofilm activity of RSE, we hypothesized that RSE might interfere with bacterial motility to inhibit early‐stage biofilm formation.

**FIGURE 2:**
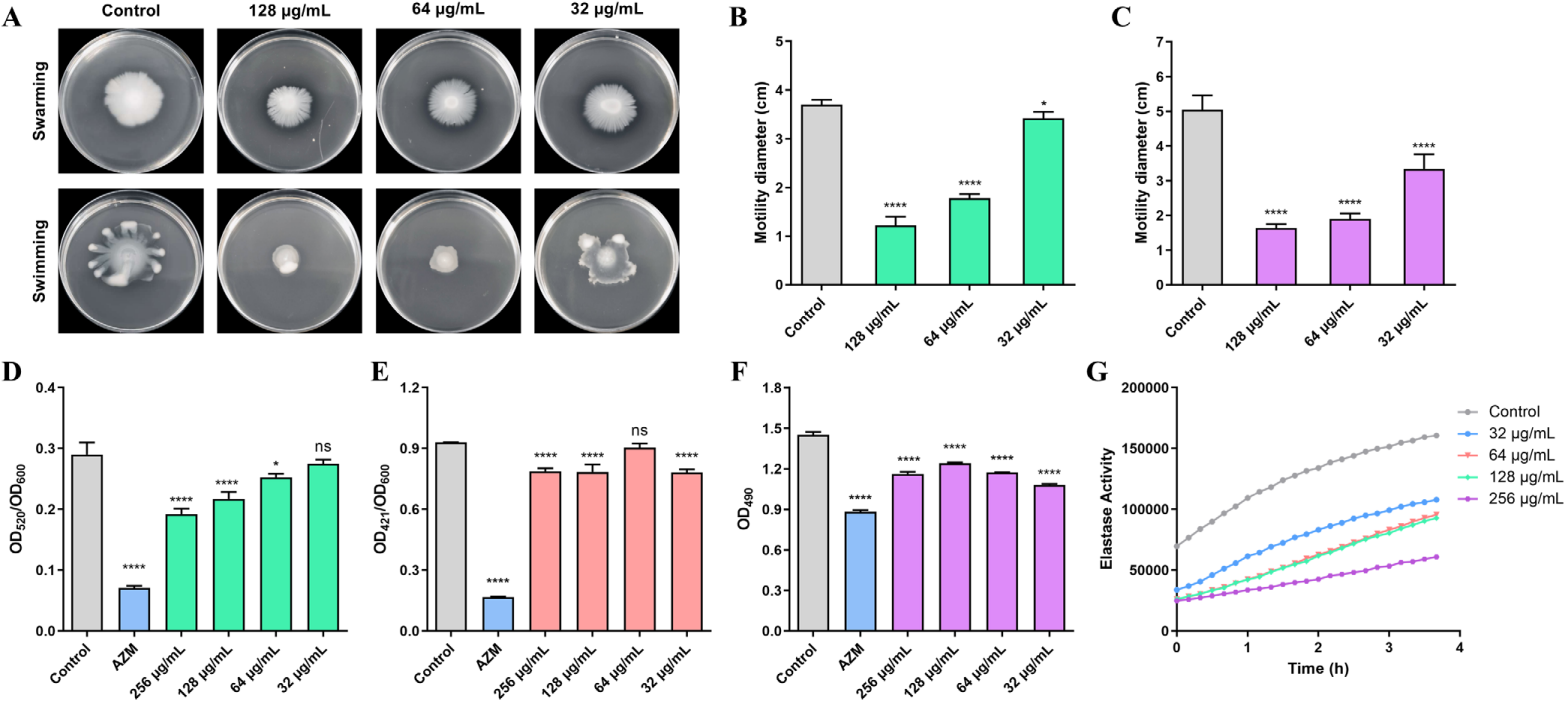
Inhibitory activity of RSE on the swarming and swimming motility and on virulence factors of PAO1. (A–C) Swarming and swimming motility zones after treatment with RSE (32–128 μg/mL). (D) Pyocyanin production. (E) Rhamnolipid production. (F) Extracellular polysaccharide (EPS) production. (G) Elastase activity measured by fluorescence over time. Different baseline fluorescence intensities at time zero reflect the inhibitory effect of RSE on elastase production/secretion during the 24 h pre‐incubation period. AZM: Azithromycin (positive control, 32 μg/mL). All data are presented as mean ± SD from three independent biological replicates (*n* = 3). Statistical significance was determined by one‐way ANOVA followed by Tukey's post hoc test. *p < 0.05, ****p < 0.0001 compared to the control.

To test this, we evaluated the effects of RSE on PAO1 swimming and swarming motility using semi‐solid agar assays. Sub‐inhibitory concentrations of RSE (≤ 128 μg/mL) were selected to exclude growth‐related confounding effects. Swarming motility assays revealed dose‐dependent inhibition by RSE (Figure 2A,B), with 128 μg/mL RSE reducing the swarming diameter by 75.21% (*p* < 0.001) compared to untreated controls.

Similarly, swimming motility was significantly suppressed (Figure 2C), with a 77.07% reduction at 128 μg/mL (*p* < 0.001). These concentrations correspond to those that inhibited biofilm formation (Section 3.1), suggesting a mechanistic link between motility impairment and anti‐biofilm activity.

These results indicate RSE impairs early‐stage colonization by targeting motility, a process intertwined with QS regulation. Since motility and QS‐linked virulence factors (e.g., elastase, pyocyanin) share upstream regulatory networks, this inhibition provides a basis for exploring RSE's effects on downstream virulence factor production, as described in the Section 3.3.

### 3.3 Inhibition of Virulence Factors Against *P. aeruginosa* PAO1

*P. aeruginosa* employs quorum sensing (QS) systems to coordinate the secretion of virulence factors critical for pathogenesis, including pyocyanin (*pqs* system), elastase (*las* system), and rhamnolipids (*rhl* system) (Singh et al. 2021; Vadakkan et al. 2024). Given RSE's observed effects on motility and biofilm formation, we hypothesized it might disrupt QS‐dependent virulence factor production. RSE significantly reduced pyocyanin and elastase secretion in a dose‐dependent manner (Figure 2D,G). Notably, 256 μg/mL RSE suppressed elastase activity by 77.07%, aligning with its strong inhibition of *las* system‐regulated phenotypes. Of note, the fluorescence intensity at time zero was already lower in RSE‐treated groups compared to the control, indicating that RSE suppressed elastase production or activity during the initial 24 h culture period. This baseline reduction was followed by a slower increase in fluorescence over time, consistent with the overall inhibition of elastase activity. In contrast, rhamnolipid production showed minimal response to RSE (Figure 2E), suggesting preferential targeting of the *las/pqs* systems. These findings support RSE's potential as a QS inhibitor, particularly against the *las* system, which is central to virulence factor regulation.

The observed suppression of elastase (LasB) and pyocyanin (PqsA) production provides a mechanistic basis for exploring RSE's effects on QS pathway activation. To further validate this, we employed fluorescent reporter strains (PAO1 *lasB*‐*gfp* and PAO1 *pqsA*‐*gfp*) to directly monitor *las* and *pqs* system activity, as described in the following section.

### 3.4 Inhibition of the RSE on *P. aeruginosa* QS Pathway

The QS network of *P. aeruginosa* coordinates virulence factor production through three interconnected systems: *las* (regulating elastase via *lasB*), *rhl* (controlling rhamnolipid synthesis via *rhlA*), and *pqs* (governing pyocyanin production via *pqsA*) (Vadakkan et al. 2024). To pinpoint the molecular targets of RSE within these pathways, we employed GFP reporter strains to monitor system‐specific activity, alongside a PAO1 *gfp* control to exclude non‐specific effects on GFP expression.

**FIGURE 3:**
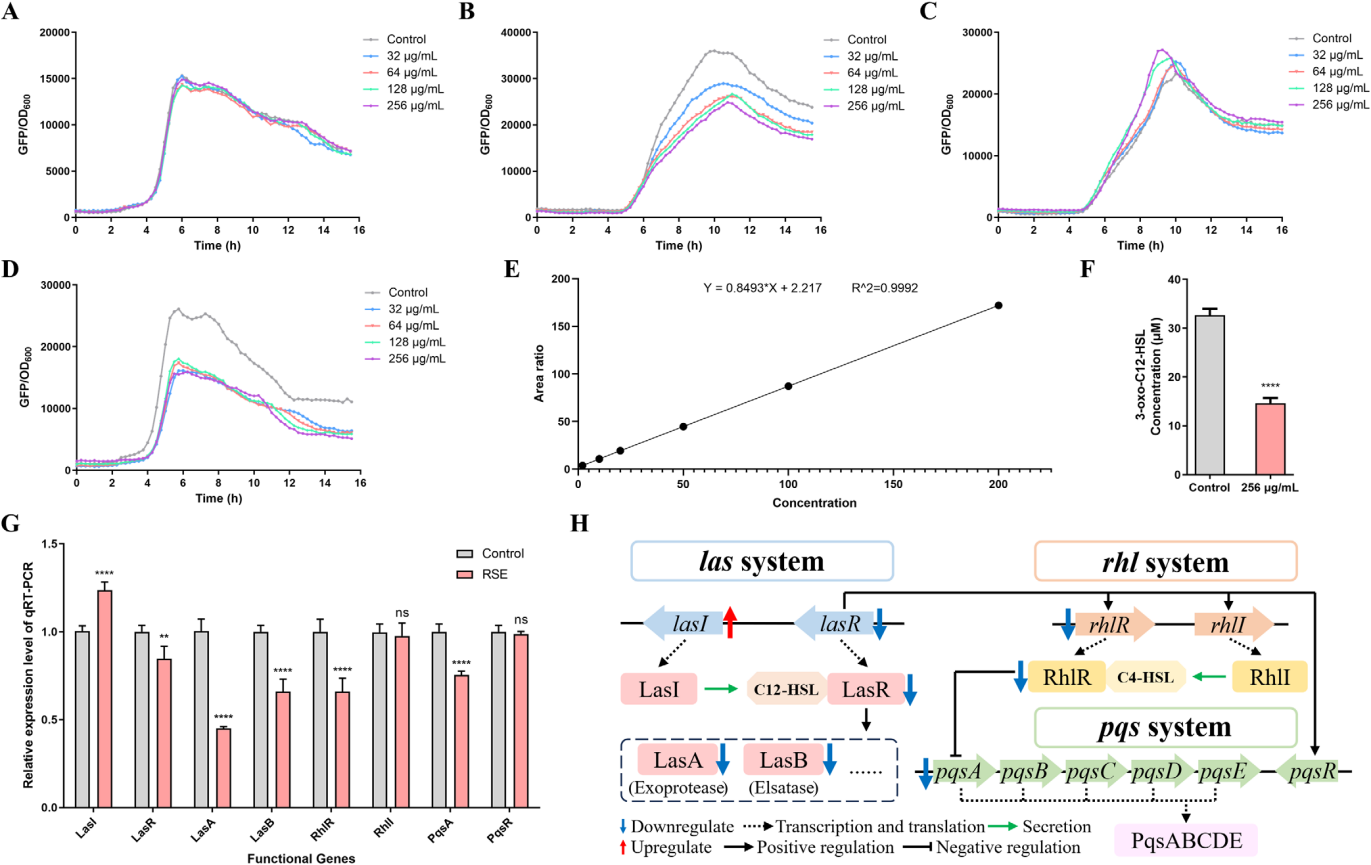
Inhibition of RSE on *P. aeruginosa* QS pathways. (A–D) Fluorescence kinetics of reporter strains: (A) PAO1 *gfp*, (B) PAO1 *lasB‐gfp*, (C) PAO1 *pqsA‐gfp* and (D) PAO1 *rhlA‐gfp*. Data are normalized to control and shown as mean ± SD (*n* = 3 biological replicates). (E) Standard curve of 3‐oxo‐C12‐HSL. (F) Quantification of 3‐oxo‐C12‐HSL after RSE treatment. Data are mean ± SD (*n* = 3). One‐way ANOVA with Tukey's post hoc test. (G) qRT‐PCR analysis of QS‐related gene expression (normalized to 16S rRNA). Data are mean ± SD (*n* = 3). One‐way ANOVA with Tukey's post hoc test. **p < 0.01, ****p < 0.0001 compared to the control.

Within the *las* quorum sensing system, the lasB gene encodes elastase, while in the *rhl* system, rhlA directs rhamnolipid biosynthesis. The *pqs* system utilizes *pqsA* for pyocyanin production, completing this virulence factor regulatory network. To elucidate the molecular targets of RSE in *P. aeruginosa* QS systems, we employed three reporter strains (PAO1 *lasB‐gfp*, PAO1 *rhlA‐gfp*, and PAO1 *pqsA‐gfp*) alongside a GFP control strain (PAO1 *gfp*). As evidenced in Figure 3B, RSE treatment significantly reduced GFP fluorescence intensity in the PAO1 *lasB‐gfp* reporter strain in a dose‐dependent manner, demonstrating its specific inhibitory effect on LasB elastase expression. Furthermore, significant *pqsA* suppression was also observed (Figure 3D), while PAO1 *rhlA‐gfp* fluorescence remained unaffected across all concentrations tested (Figure 3C). These molecular‐level findings align with our phenotypic observations of reduced elastase and pyocyanin production, establishing that RSE specifically inhibits the *las* and *pqs* system with negligible effects on the *rhl* system.

The *las* quorum sensing system in *P. aeruginosa* is governed by two core components: the transcriptional regulator LasR and the synthase LasI (Mukherjee et al. 2017; Turkina and Vikström 2018). LasI catalyzes the production of the autoinducer *N*‐(3‐oxododecanoyl)‐L‐homoserine lactone (3‐oxo‐C12‐HSL), which binds LasR to form an active transcriptional complex that regulates virulence gene expression (Mukherjee et al. 2018). To investigate RSE's interference with this signaling pathway, we quantified 3‐oxo‐C12‐HSL levels in bacterial cultures using UPLC‐Q‐Orbitrap‐MS/MS with umbelliferone as an internal standard. RSE treatment induced a concentration‐dependent reduction in 3‐oxo‐C12‐HSL production (Figure 3F), with 256 μg/mL RSE causing a 59.38% decrease. This significant suppression of AHL secretion confirms that RSE specifically targets the *las* system, corroborating our earlier observations of diminished virulence factor production.

The *las* system utilizes 3‐oxo‐C12‐HSL (synthesized by LasI) to activate LasR, upregulating virulence genes (*lasA*, *lasB*, exotoxins). Similarly, the *rhl* system employs RhlI‐produced C4‐HSL to stimulate RhlR‐mediated gene expression, while the *pqs* system generates PQS (*pqsABCDE*‐encoded) that binds PqsR to control pyocyanin synthesis and eDNA release for biofilm development (Vadakkan et al. 2024). To further study the molecular mechanisms underlying the anti‐virulence effects, we analyzed the expression level of key QS components in *P. aeruginosa* PAO1 following RSE treatment using qRT‐PCR. Consistent with the reporter strain results, qRT‐PCR further validated that RSE treatment significantly downregulated the expression of several key QS‐related genes (Figure 3G), with particularly moderate effects on *lasA* (55% reduction) and *lasB* (42%), while the *pqs* system genes *pqsA* were downregulated by approximately 25%. Interestingly, while *lasI* transcript levels showed a modest increase (approximately 24% above control), the expression of *lasR*, *lasA*, and *lasB* was reduced. This discordant pattern—upregulation of the autoinducer synthase gene but downregulation of the receptor and downstream effector genes—suggests that RSE disrupts the *las* QS pathway at a level beyond simple transcriptional repression, possibly by interfering with signal molecule accumulation or receptor function. This interpretation is supported by the observed reduction in 3‐oxo‐C12‐HSL levels (Figure 3F). These findings demonstrate RSE's suppression of the *las* QS cascades, consistent with its observed phenotypic inhibition of virulence factor production and biofilm formation.

### 3.5 Qualitative Analysis of RSE

**FIGURE 4:**
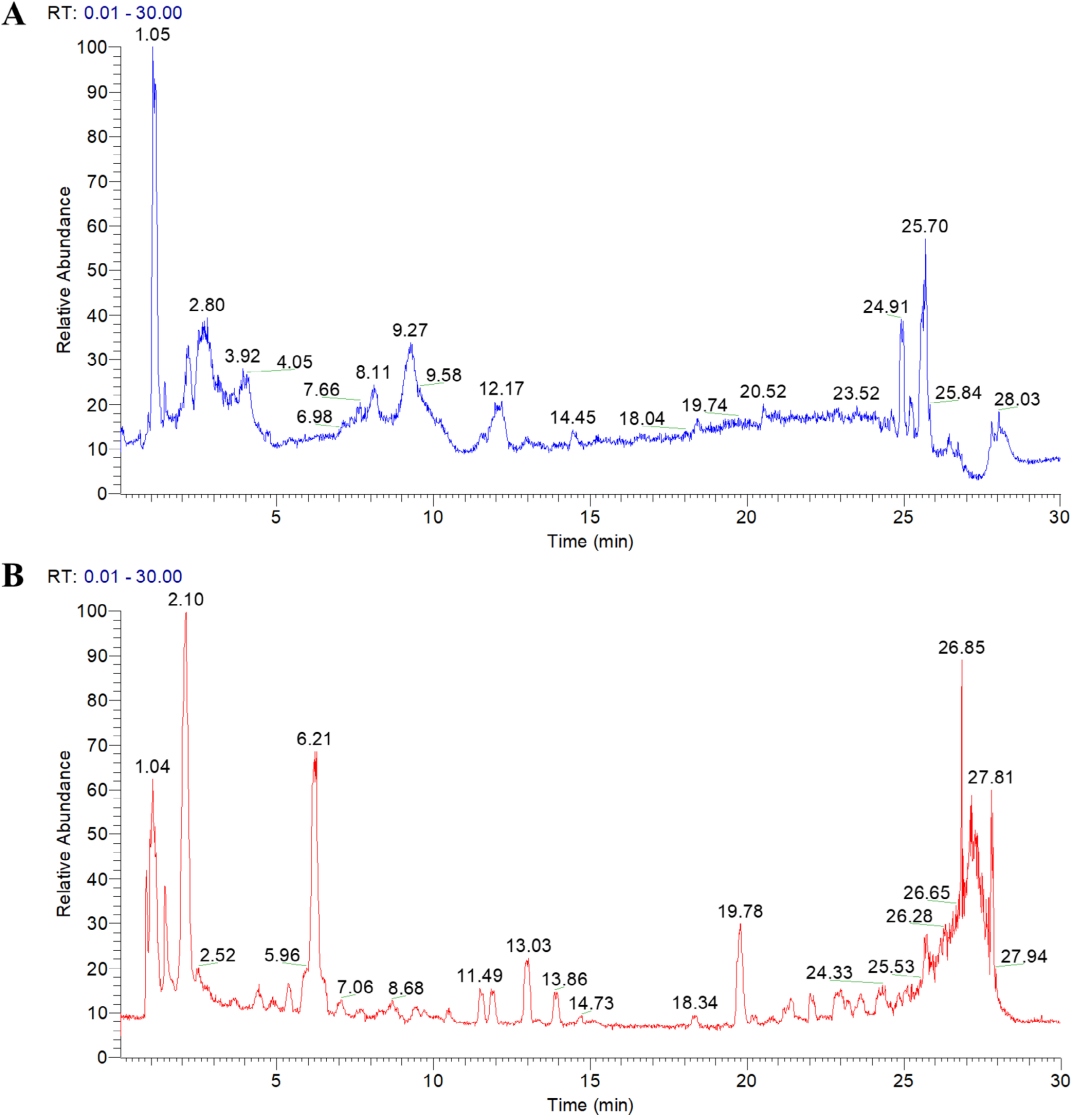
TIC diagram of Raphani Semen extract UPLC‐Q‐Orbitrap‐MS/MS: (A) negative, (B) positive.

The primary constituents of RS include glucosinolates, isothiocyanates, sinapic acid derivatives, flavonoids, fatty acids, and amino acids, among which glucosinolates and sinapic acid compounds have been extensively studied in recent years (Gao et al. 2022). To elucidate the bioactive components responsible for inhibiting the *las* quorum sensing system in *P. aeruginosa* , we performed qualitative phytochemical analysis of RSE using UPLC‐Q‐Orbitrap‐MS/MS. The total ion chromatogram (TIC) of RSE was shown in Figure 4. A total of 71 compounds were identified (Figure 4), classified as 12 glucosinolates, 6 isothiocyanates, 15 phenolic acids (including 6 sinapine derivatives), 15 sinapic acid esters, 7 flavonoids, and 16 additional metabolites (Figure S1 and Tables S3 and S4). The identity of each compound was confirmed by comparison of its MS/MS fragmentation pattern with reference spectra. All MS/MS spectra are provided in Figure S7–S77. Structural elucidation was achieved through retention time alignment and multistage mass spectrometry fragmentation patterns, providing comprehensive characterization of RSE components potentially targeting the *P. aeruginosa* *las* system.

Based on our UPLC‐Q‐Orbitrap‐MS/MS profiling which identified a range of glucosinolates and isothiocyanates in RSE, we selected two characteristic isothiocyanates, SFA and SFE, for further investigation. These two compounds are not the most abundant constituents in RSE. Instead, they were chosen because: (i) isothiocyanates (their chemical class) are widely reported to possess anti‐quorum sensing activity; (ii) both are commercially available with documented anti‐QS effects in other bacterial systems, allowing us to test whether this class contributes to RSE's activity; and (iii) they are characteristic isothiocyanates of Raphani Semen, supporting their relevance as representative markers. Despite their moderate abundance, SFA and SFE were thus selected to investigate the potential contribution of isothiocyanates to the anti‐QS activity of RSE. We therefore aimed to evaluate their contribution to the anti‐virulence activity observed with the crude extract.

### 3.6 MIC and Biofilm Formation Inhibition of SFA and SFE

Broth microdilution assays showed that SFA and SFE did not inhibit bacterial growth at concentrations up to 1000 μM (the highest concentration tested), yielding MIC values > 1000 μM for both compounds (Table S2). Higher concentrations were not tested due to limited pharmacological relevance. Thus, the sub‐inhibitory concentrations used in subsequent experiments (12.5–100 μM) were well below the MIC.

The anti‐biofilm activity of both compounds was confirmed using crystal violet staining. SFA and SFE inhibited biofilm formation in a dose‐dependent manner (Figure S1). At 50 μM, both SFA and SFE reduced biofilm biomass by approximately 50%, an inhibition level comparable to that achieved by the positive control, 32 μg/mL azithromycin. At the highest tested concentration of 100 μM, SFA and SFE further suppressed biofilm formation by 67.8% and 52.4%, respectively. CLSM imaging further supported these findings (Figure S2C–H), showing clear reductions in biofilm thickness and structural complexity. These results establish SFA and SFE as key active constituents underlying the anti‐biofilm properties of RSE.

To evaluate the potential of SFA and SFE relative to conventional antibiotics, we compared their anti‐biofilm efficacy with azithromycin and assessed their synergistic interactions with four clinical antibiotics (azithromycin, amikacin, ceftazidime, and cefixime). The fractional inhibitory concentration index (FICI) was calculated using the checkerboard microdilution method. The detailed results of these synergy assays, including FICI values and interpretations, are summarized in Table 1. The results showed that SFA acted synergistically with amikacin (FICI = 0.3) and ceftazidime (FICI = 0.128). SFE also showed synergy with ceftazidime (FICI = 0.3) and an additive effect with amikacin (FICI = 0.6). Collectively, these comparisons with conventional antibiotics demonstrate that SFA and SFE not only exhibit anti‐biofilm efficacy comparable to azithromycin but also possess adjuvant potential to enhance the activity of existing antibiotics.

**TABLE 1:** Synergistic and additive effects of SFA and SFE combined with four antibiotics against PAO1.

Compound	Antibiotic	FICI*	Result
SFA	Azithromycin	1.2	Indifference
	Amikin	0.3	Synergy
	Cefixime	1.2	Indifference
	Ceftazidime	0.128	Synergy
SFE	Azithromycin	1.2	Indifference
	Amikin	0.6	Additivity
	Cefixime	1.2	Indifference
	Ceftazidime	0.3	Synergy

* Interpretation of the FICI results were as follows: ≤ 0.5, synergy; 0.5–1.0, additivity; > 1.0–4.0, indifference; and > 4, antagonism.

### 3.7 Influence of SFA and SFE on Swimming and Swarming Motility

The effects of SFA and SFE on *P. aeruginosa* motility were also assessed, revealing a consistent pattern with that observed for RSE. Both compounds suppressed swarming and swimming motility in a dose‐dependent manner (Figure S3A–D), mirroring the anti‐motility effects of the crude extract. At 100 μM, SFA reduced swarming and swimming by 57.9% and 49.3%, while SFE decreased these motility phenotypes by 55.5% and 53.8%.

Similarly, pyocyanin production was significantly attenuated by both monomers in a concentration‐dependent manner (Figure S3E), paralleling the strong inhibition previously seen with RSE. Treatment with 100 μM SFA and SFE inhibited pyocyanin secretion by 62.6% and 42.3%, correspondingly. In contrast, rhamnolipid production was only slightly reduced by SFA at higher concentrations, and SFE showed no detectable effect (Figure S3F). This selective inhibition profile—potently suppressing pyocyanin while minimally affecting rhamnolipids—aligns with and potentially explains the earlier observations for RSE. Notably, both compounds also markedly inhibited extracellular polysaccharide (EPS) synthesis (Figure S3G). At 100 μM, SFE showed stronger suppression of EPS (65.6%) than SFA (38.2%).

Taken together, these results indicate that SFA and SFE, at sub‐inhibitory concentrations, recapitulate and help explain the selective anti‐virulence profile of RSE, particularly targeting pyocyanin production and EPS synthesis while showing minimal effects on rhamnolipid production.

### 3.8 Inhibition of the SFA and SFE on *P. aeruginosa* QS Pathway

**FIGURE 5:**
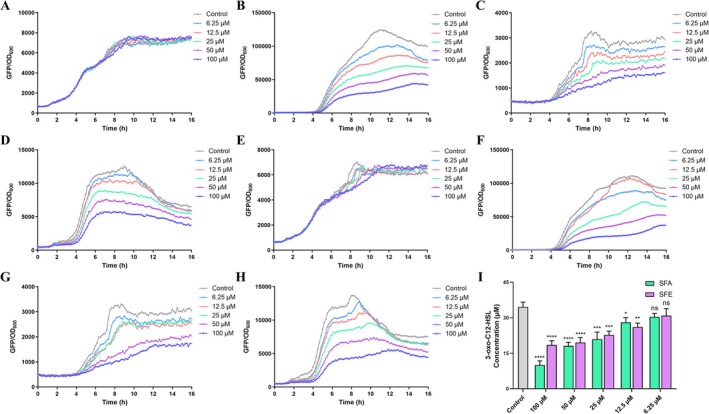
Inhibition curves of SFA and SFE on QS reporter strains and 3‐oxo‐C12‐HSL production. (A–D) SFA: (A) PAO1 *gfp*, (B) PAO1 *lasB‐gfp*, (C) PAO1 *rhlA‐gfp*, (D) PAO1 *pqsA‐gfp*. (E–H) SFE: (E) PAO1 *gfp*, (F) PAO1 *lasB‐gfp*, (G) PAO1 *rhlA‐gfp*, (H) PAO1 *pqsA‐gfp*. Fluorescence data are presented as mean ± SD from three independent biological replicates (*n* = 3). (I) Quantification of 3‐oxo‐C12‐HSL after treatment with SFA or SFE. Data are mean ± SD (*n* = 3). One‐way ANOVA with Tukey's post hoc test. *p < 0.05, **p < 0.01, ***p < 0.001, ****p < 0.0001 compared to the control.

As shown in Figure 5A–H, both SFA and SFE markedly diminished GFP fluorescence in all three QS reporter strains in a dose‐dependent manner. These results confirm that the two compounds effectively suppress the expression of key QS genes (*lasB* and *pqsA*), thereby interfering with the *las* and *pqs* systems of *P. aeruginosa* . Interestingly, although phenotypic assays showed only marginal inhibition of rhamnolipid secretion by SFA at high concentrations and no detectable effect for SFE, the PAO1 *rhlA*‐*gfp* reporter strain revealed significant suppression of *rhlA* gene expression by both compounds (Figure 5C,G). This apparent discrepancy between gene expression and metabolic output may be attributed to various factors, including post‐transcriptional regulatory mechanisms and the redundancy and compensatory pathways inherent in bacterial metabolic networks, which can maintain secondary metabolite production despite reduced gene expression levels. These findings highlight the complexity of QS‐regulated virulence factor production. Overall, the results for SFA and SFE align with and substantiate those observed for RSE, supporting their role as effective inhibitors targeting the *las* and *pqs* systems.

To further elucidate the mechanism of *las* system interference, we quantified the production of the key signal molecule 3‐oxo‐C12‐HSL. Both compounds significantly reduced its secretion in bacterial cultures (Figure 5I). At 100 μM, SFA suppressed production by 74.29%, and SFE by 51.43%, revealing a clear dose–response relationship. This observation is consistent with the inhibitory effects observed in the *lasB*‐*gfp* reporter strain. The PAO1 *lasB‐gfp* reporter strain showed a delayed increase in fluorescence (starting at approximately 4 h; Figure 5B,F), which is characteristic of Las‐dependent promoters that require accumulation of the autoinducer 3‐oxo‐C12‐HSL to a threshold concentration. The other reporter strains (*rhlA‐gfp* and *pqsA‐gfp*) exhibited different kinetic profiles due to their distinct regulatory circuits, as described previously. These findings confirm that the QS‐inhibitory activity of the crude RSE extract can be largely attributed to these two constituents, which function primarily through suppression of the upstream *las* system and downstream *pqs* system signaling pathways.

### 3.9 Molecular Docking

**FIGURE 6:**
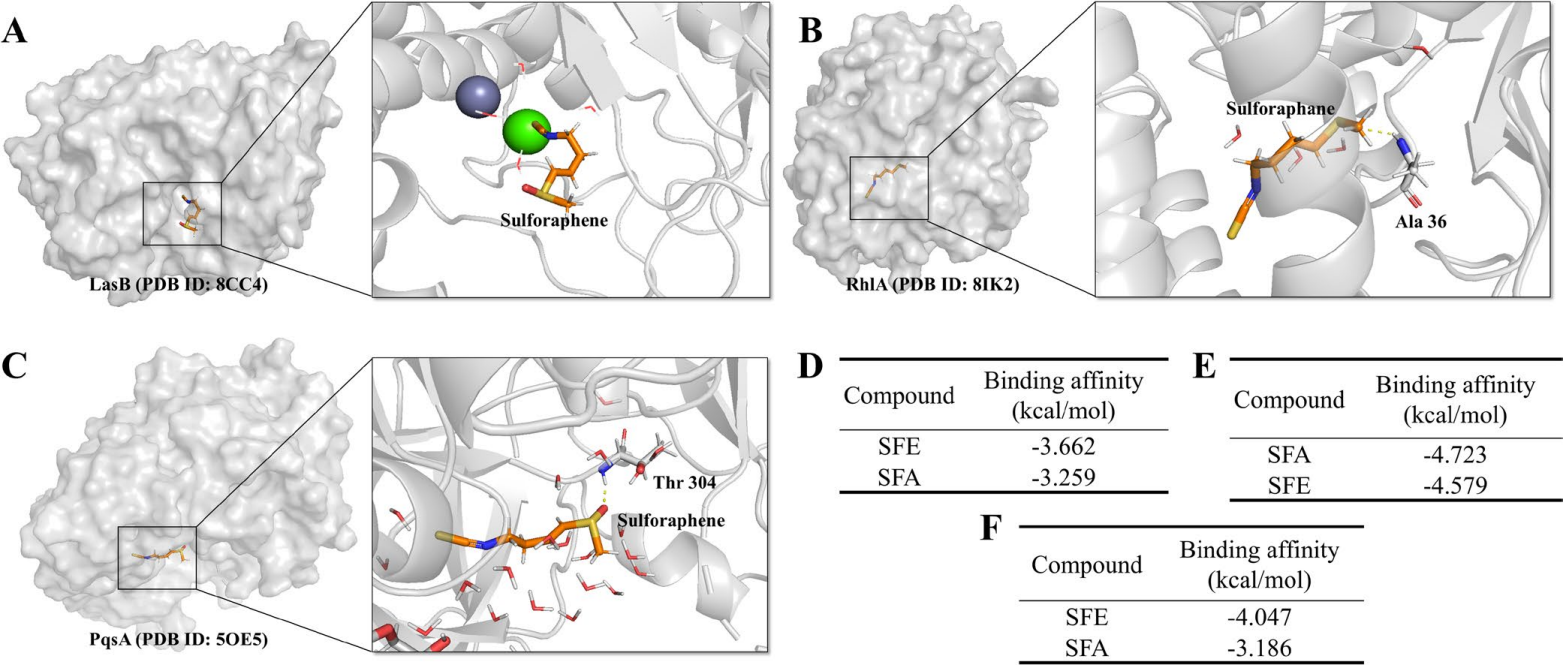
Molecular docking analysis of SFA and SFE with QS target proteins. (A) Binding mode of SFE with LasB (PDB: 8CC4). (B) Interaction of SFA with RhlA (PDB: 8IK2); hydrogen bond with Ala 36 is highlighted. (C) Interaction of SFA with PqsA (PDB: 5OE5); hydrogen bond with Thr 304 is highlighted. (D–F) Calculated binding affinities (kcal/mol) for LasB (D), RhlA (E), and PqsA (F). Docking scores represent a single calculation per protein‐ligand pair; no statistical analysis was performed.

To provide structural insights into the mechanism by which RSE active components inhibit QS systems, molecular docking was employed to simulate the interactions of SFA and SFE with LasB, RhlA, and PqsA. The binding affinities (docking scores) are summarized in Figure 6D–F. Generally, negative binding energies indicate spontaneous interactions, with lower values representing more stable complexes.

For the *las* system, the crystal structure of the elastase LasB (PDB ID: 8CC4) was used as the target. As shown in Figure 6D, both compounds exhibited binding potential, with SFE showing a slightly stronger affinity (−3.662 kcal/mol) compared to SFA (−3.259 kcal/mol). The docking pose (Figure 6A) reveals that SFE occupies the active site pocket of LasB, positioning itself in close proximity to the zinc metalloprotease catalytic center, which potentially hinders substrate access and explains the reduced elastase activity observed in vitro.

In the *rhl* system, despite the modest phenotypic inhibition of rhamnolipids, docking results with RhlA (PDB ID: 8IK2) showed the strongest binding affinities among the tested proteins (Figure 6E). SFA exhibited the highest binding energy of −4.723 kcal/mol, while SFE showed −4.579 kcal/mol. Visual inspection of the SFA‐RhlA complex (Figure 6B) demonstrates a key hydrogen bond interaction with residue Ala 36, stabilizing the ligand within the binding groove. This suggests that SFA and SFE have the structural capacity to bind RhlA, aligning with the transcriptional downregulation of the rhlA gene (Section 3.8).

Regarding the *pqs* system, PqsA (PDB ID: 5OE5) is critical for PQS signal synthesis. Docking analysis (Figure 6F) indicated that SFE binds more tightly (−4.047 kcal/mol) than SFA (−3.186 kcal/mol). The binding mode of SFE (Figure 6C) highlights a specific hydrogen bond interaction with Thr 304, a residue located within the active site tunnel involved in anthranilate binding. This physical occlusion likely interferes with the enzymatic conversion of anthranilate to anthraniloyl‐CoA, consistent with the observed reduction in pyocyanin production.

Collectively, these molecular docking simulations support the experimental findings, suggesting that SFA and SFE can bind to LasB, RhlA, and PqsA, albeit with modest affinities (docking scores ranging from −3.2 to −4.5 kcal/mol). This suggests that the compounds may have the potential to interact with multiple QS‐related targets, consistent with their moderate inhibitory effects on the corresponding phenotypic outputs.

## 4 Discussion

This study demonstrates that RSE and its isothiocyanate constituents SFA and SFE inhibit *P. aeruginosa* biofilm formation and virulence primarily by disrupting the *las* quorum sensing system. Unlike conventional antibiotics that kill bacteria or stop their growth, quorum sensing inhibitors suppress virulence factor production and biofilm formation without exerting strong selective pressure, thereby reducing the risk of resistance development (Lu et al. 2022; Maiga et al. 2025). The present findings add to the growing list of natural products that target QS and, importantly, identify a medicinal and edible plant, Raphani Semen, as a source of anti‐QS isothiocyanates.

The upstream position of the *las* system in the *P. aeruginosa* QS hierarchy makes it an attractive target for anti‐virulence strategies (Vadakkan et al. 2024). In this study, RSE reduced the production of the *las*‐specific autoinducer 3‐oxo‐C12‐HSL by up to 59% and downregulated *lasR* and *lasI* expression. The concomitant decrease in elastase (LasB) and pyocyanin is therefore a downstream consequence of *las* inhibition, not direct targeting of these enzymes. This mechanism resembles that of other plant‐derived QSIs, such as curcumin, which reduces AHL production (Shukla et al. 2020). However, unlike many broad‐spectrum QSIs, RSE showed negligible effect on the *rhl* system. This narrower specificity may be therapeutically advantageous by minimizing off‐target effects on beneficial microbiota.

The purified monomers SFA and SFE reproduced the key anti‐biofilm and anti‐virulence effects of RSE: they reduced 3‐oxo‐C12‐HSL, inhibited *lasB‐gfp* and *pqsA‐gfp* reporter activity, and suppressed pyocyanin and extracellular polysaccharide production. These results confirm that isothiocyanates contribute to the QS‐inhibitory activity of RSE, consistent with previous reports on the anti‐QS properties of broccoli‐derived sulforaphane (Ganin et al. 2012; Bendary et al. 2024).

A striking observation was that the monomers exhibited broader activity than the crude extract: unlike RSE, SFA and SFE also suppressed *rhlA‐gfp* transcription. Several explanations may account for this difference. First, the crude extract may contain components that antagonize or buffer the effect of isothiocyanates on the *rhl* system, a phenomenon documented in other herbal mixtures (Wen et al. 2024). Second, the monomers might be more bioavailable or less protein‐bound in their pure form, allowing them to reach and inhibit pathways that remain less affected by the extract. Third, a concentration‐dependent threshold for *rhl* inhibition may exist; RSE was tested at 32–256 μg/mL, while the monomers were tested up to 100 μM.

Both SFA and SFE significantly reduced *rhlA‐gfp* reporter fluorescence, yet rhamnolipid production was not significantly changed. This apparent discrepancy deserves careful interpretation. Rhamnolipid synthesis is a multi‐step process requiring three enzymes: RhlA, RhlB, and RhlC. Transcriptional downregulation of *rhlA* alone may not limit final product formation if RhlB and RhlC remain active or if residual RhlA activity suffices (Déziel et al. 2003; Vadakkan et al. 2024). Second, post‐transcriptional and post‐translational regulatory mechanisms, as well as feedback loops within the *rhl* system, can compensate for reduced transcript levels (Mukherjee et al. 2017; Turkina and Vikström 2018). Finally, the *rhlA‐gfp* reporter, being a fusion construct, may not fully recapitulate the regulation of the native *rhlA* gene due to the absence of certain regulatory elements. Despite this inconsistency, the consistent inhibition of the *las* and *pqs* systems and the robust anti‐biofilm activity remain unchanged.

Molecular docking gave calculated binding energies for SFA and SFE with LasB, RhlA, and PqsA ranging from −3.2 to −4.5 kcal/mol, which are generally considered weak to moderate. However, weak binding is not unexpected for small, moderately hydrophobic isothiocyanates. Moreover, quorum sensing inhibitors often achieve their effects at sub‐MIC concentrations through multiple low‐affinity interactions rather than a single high‐affinity binding event (Ganin et al. 2012; Bendary et al. 2024). The fact that these modest affinities were observed consistently across three different target proteins supports the concept of a multi‐target mechanism. Nevertheless, the docking results are predictive and require experimental validation, for example by surface plasmon resonance or direct enzyme activity assays.

From a food science and nutrition perspective, Raphani Semen is officially listed as a “medicinal and edible” substance in China. Its seeds are rich in unsaturated fatty acids, vitamins B1 and B2, polysaccharides, and sinapine derivatives, and have been consumed as a functional food ingredient. The isothiocyanates SFA and SFE are also naturally present in common cruciferous vegetables such as broccoli, radish sprouts, and cabbage. Thus, the anti‐biofilm and anti‐virulence activities described here have direct relevance to food science. First, RSE and its isothiocyanates could be developed as natural food preservatives, as QS inhibitors reduce surface contamination without killing bacteria, thereby avoiding the development of preservative resistance. Second, the synergy observed between SFA/SFE and antibiotics (amikacin, ceftazidime) suggests that isothiocyanate‐rich RSE could serve as dietary adjuvants to enhance antibiotic efficacy, particularly in gut or respiratory infections. Third, given that *P. aeruginosa* is an opportunistic pathogen associated with gastrointestinal disorders, incorporating RSE or its isothiocyanates into functional foods or nutraceuticals might offer a complementary strategy for infection management without disrupting the gut microbiota—a key advantage over broad‐spectrum antibiotics.

Several limitations should be acknowledged. All experiments were performed with the wild type strain PAO1; activity against clinical multidrug‐resistant isolates has not been evaluated. The study is entirely in vitro; in vivo efficacy in animal infection models, along with pharmacokinetic evaluation, is required. For the monomers SFA and SFE, qRT‐PCR was not performed; transcriptional inhibition was inferred from reporter strains. The docking scores are weak and require experimental validation. The long‐term stability of RSE and its constituents under physiological conditions, as well as their biocompatibility towards mammalian cells, has not been characterized. Finally, while SFA and SFE were identified as active constituents, the extract contains 71 compounds; synergistic or antagonistic interactions among other constituents warrant further investigation.

Despite these limitations, this study provides a mechanistic basis for the anti‐virulence activity of Raphani Semen and identifies its isothiocyanate components as promising QS inhibitors. The differential effects between the crude extract and pure compounds offer a valuable lesson in natural product research: the activity of a monomer does not always predict the activity of the complex mixture, and vice versa. By targeting bacterial communication rather than bacterial survival, RSE and its isothiocyanates represent a novel paradigm for managing *P. aeruginosa* infections in both clinical and food‐related settings.

## 5 Conclusion

In this study, phytochemical characterization via UPLC‐Q‐Orbitrap‐MS/MS identified 71 compounds in RSE. Among these constituents, SFA and SFE were identified as the key active components responsible for the observed QS inhibitory effects against *P. aeruginosa* PAO1. Our findings demonstrate that both SFA and SFE specifically target the *las* QS system, significantly suppressing key virulence phenotypes including biofilm formation, bacterial motility, and the production of virulence factors and the signal molecule 3‐oxo‐C12‐HSL. At the molecular level, these phenotypic effects correlate with the downregulation of *las* system genes and inhibition of their promoter activities, as confirmed by QS reporter strain assays. Collectively, these results position RSE and its active components, SFA and SFE, as promising candidates for novel anti‐virulence strategies. They offer a viable approach to combat *P. aeruginosa* pathogenicity by disrupting its communication and virulence networks, thereby potentially mitigating the development of conventional antibiotic resistance.

## Author Contributions

**Jun Xu:** software, resources, visualization. **Honggeng Chen:** data curation, formal analysis, investigation, methodology. **Hongxia Fan:** project administration, supervision, validation. **Xin Yu:** supervision, validation, writing – review and editing. **Jianni Yang:** writing – review and editing, validation, supervision. **Yujing Huang:** data curation, software, formal analysis, writing – original draft. **Zhiyun Xia:** data curation, formal analysis, visualization, writing – original draft. **Juntao Xie:** data curation, formal analysis, visualization, validation, writing – original draft. **Likang Wang:** data curation, formal analysis. **Junxia Zheng:** conceptualization, funding acquisition, project administration, resources, supervision, validation, writing – review and editing.

## Funding

This work was supported by the Guangdong Basic and Applied Basic Research Foundation (2023A1515011445, 2022A1515220142), the National Natural Science Foundation of China (No.82574239), and the Science and Technology Projects in Guangzhou (2024A04J6491).

## Conflicts of Interest

The authors declare no conflicts of interest.

## Supporting information


**Figure S1:** Analysis and summary of chemical composition of RSE. The figure shows the classification of the 71 compounds identified by UPLC‐Q‐Orbitrap‐MS/MS, including glucosinolates (12), isothiocyanates (6), phenolic acids (15), sinapic acid esters (15), flavonoids (7), and other metabolites (16).
**Figure S2:** Inhibition of biofilm formation by SFA and SFE. (A, B) Biofilm biomass measured by crystal violet staining after treatment with increasing concentrations of SFA (A) or SFE (B). AZM: azithromycin (positive control, 32 μg/mL). (C, D) COMSTAT analysis of total biofilm biomass for SFA (C) and SFE (D). (E, F) COMSTAT analysis of average biofilm thickness for SFA (E) and SFE (F). (G, H) Representative CLSM images of 24 h biofilms treated with SFA (G) or SFE (H). All data are presented as mean ± SD from three independent biological replicates (*n* = 3). Statistical significance was determined by one‐way ANOVA followed by Tukey's post hoc test. *p < 0.05, **p < 0.01, ***p < 0.001, ****p < 0.0001 compared to the control.
**Figure S3:** Effects of SFA and SFE on motility and virulence factors. (A–D) Swimming and swarming motility zones after treatment with SFA (A, B) or SFE (C, D). (E–G) Pyocyanin (E), rhamnolipid (F) and extracellular polysaccharide (G) production after SFA treatment. (H–J) Pyocyanin (H), rhamnolipid (I) and extracellular polysaccharide (J) production after SFE treatment. AZM: azithromycin (positive control, 32 μg/mL). Data are presented as mean ± SD from three independent biological replicates (*n* = 3). One‐way ANOVA with Tukey's post hoc test was used. *p < 0.05, **p < 0.01, ***p < 0.001, ****p < 0.0001 compared to the control.
**Figure S4:** Agarose gel electrophoresis of PCR products. PCR products for the target genes (16S rRNA, lasI, lasR, lasA, lasB, rhlI, rhlR, pqsR, pqsA) were separated on a 1.5% agarose gel. Lane M: DNA marker (e.g., 100 bp ladder). The expected amplicon sizes are listed in Table 1.
**Figure S5:** Amplification curves (primary curves) of qRT‐PCR. (A) *16S rRNA*, (B) *lasI*, (C) *lasR*, (D) *lasA*, (E) *lasB*, (F) *rhlI*, (G) *rhlR*, (H) *pqsR*, (I) *pqsA*. Fluorescence was plotted against cycle number for each gene.
**Figure S6:** Melting curves of qRT‐PCR. (A) *16S rRNA*, (B) *lasI*, (C) *lasR*, (D) *lasA*, (E) *lasB*, (F) *rhlI*, (G) *rhlR*, (H) *pqsR*, (I) *pqsA*. The negative first derivative of fluorescence (−dF/dT) was plotted against temperature to verify the specificity of each amplicon.

**Figure S7:** The MS/MS spectrum of N1.
**Figure S8:** The MS/MS spectrum of N2.
**Figure S9:** The MS/MS spectrum of N3.
**Figure S10:** The MS/MS spectrum of N4.
**Figure S11:** The MS/MS spectrum of N5.
**Figure S12:** The MS/MS spectrum of N6.
**Figure S13:** The MS/MS spectrum of N7.
**Figure S14:** The MS/MS spectrum of N8.
**Figure S15:** The MS/MS spectrum of N9.
**Figure S16:** The MS/MS spectrum of N10.
**Figure S17:** The MS/MS spectrum of N11.
**Figure S18:** The MS/MS spectrum of N12.
**Figure S19:** The MS/MS spectrum of N13.
**Figure S20:** The MS/MS spectrum of N14.
**Figure S21:** The MS/MS spectrum of N15.
**Figure S22:** The MS/MS spectrum of N16.
**Figure S23:** The MS/MS spectrum of N17.
**Figure S24:** The MS/MS spectrum of N18.
**Figure S25:** The MS/MS spectrum of N19.
**Figure S26:** The MS/MS spectrum of N20.
**Figure S27:** The MS/MS spectrum of N21.
**Figure S28:** The MS/MS spectrum of N22.
**Figure S29:** The MS/MS spectrum of N23.
**Figure S30:** The MS/MS spectrum of N24.
**Figure S31:** The MS/MS spectrum of N25.
**Figure S32:** The MS/MS spectrum of N26.
**Figure S33:** The MS/MS spectrum of N27.
**Figure S34:** The MS/MS spectrum of N28.
**Figure S35:** The MS/MS spectrum of N29.
**Figure S36:** The MS/MS spectrum of N30.
**Figure S37:** The MS/MS spectrum of N31.
**Figure S38:** The MS/MS spectrum of N32.
**Figure S39:** The MS/MS spectrum of N33.
**Figure S40:** The MS/MS spectrum of N34.
**Figure S41:** The MS/MS spectrum of N35.
**Figure S42:** The MS/MS spectrum of N36.
**Figure S43:** The MS/MS spectrum of N37.
**Figure S44:** The MS/MS spectrum of N38.
**Figure S45:** The MS/MS spectrum of N39.
**Figure S46:** The MS/MS spectrum of N40.
**Figure S47:** The MS/MS spectrum of N41.
**Figure S48:** The MS/MS spectrum of N42.
**Figure S49:** The MS/MS spectrum of P1.
**Figure S50:** The MS/MS spectrum of P2.
**Figure S51:** The MS/MS spectrum of P3.
**Figure S52:** The MS/MS spectrum of P4.
**Figure S53:** The MS/MS spectrum of P5.
**Figure S54:** The MS/MS spectrum of P6.
**Figure S55:** The MS/MS spectrum of P7.
**Figure S56:** The MS/MS spectrum of P8.
**Figure S57:** The MS/MS spectrum of P9.
**Figure S58:** The MS/MS spectrum of P10.
**Figure S59:** The MS/MS spectrum of P11.
**Figure S60:** The MS/MS spectrum of P12.
**Figure S61:** The MS/MS spectrum of P13.
**Figure S62:** The MS/MS spectrum of P14.
**Figure S63:** The MS/MS spectrum of P15.
**Figure S64:** The MS/MS spectrum of P16.
**Figure S65:** The MS/MS spectrum of P17.
**Figure S66:** The MS/MS spectrum of P18.
**Figure S67:** The MS/MS spectrum of P19.
**Figure S68:** The MS/MS spectrum of P20.
**Figure S69:** The MS/MS spectrum of P21.
**Figure S70:** The MS/MS spectrum of P22.
**Figure S71:** The MS/MS spectrum of P23.
**Figure S72:** The MS/MS spectrum of P24.
**Figure S73:** The MS/MS spectrum of P25.
**Figure S74:** The MS/MS spectrum of P26.
**Figure S75:** The MS/MS spectrum of P27.
**Figure S76:** The MS/MS spectrum of P28.
**Figure S77:** The MS/MS spectrum of P29.
**Table S1:** Primer sequences of *lasI*, *lasR*, *lasA*, *lasB*, *rhIR*, *rhII*, *pqsA* and *pqsR*.
**Table S2:** MIC values of RSE, SFA, SFE, and azithromycin.
**Table S3:** Composition analysis of Raphani Semen extract by UPLC‐Q‐Orbitrap‐MS/MS (negative ion mode).
**Table S4:** Composition analysis of Raphani Semen extract UPLC‐Q‐Orbitrap‐MS/MS (positive ion mode).

## Data Availability

The data that support the findings of this study are available on request from the corresponding author. The data are not publicly available due to privacy or ethical restrictions.
