# Supplementary material for: Raphani Semen Extract Targets the las Quorum Sensing System to Suppress Pseudomonas aeruginosa Biofilm and Signal Molecule Production
Source: Food Sci Nutr. 2026 Aug 12;14(8):e72034. doi: 10.1002/fsn3.72034 (PMC13469746; doi:10.1002/fsn3.72034)
Supplement: Supplementary file 1 [file FSN3-14-e72034-s001.docx]

**Supplementary material_1**

**Raphani Semen Extract Targets the *las* Quorum Sensing System to Suppress *Pseudomonas aeruginosa* Biofilm and Signal Molecule Production**

Juntao Xie^1^, Yujing Huang^1,2^, Honggeng Chen^1^, Zhiyun Xia^1^, Likang Wang^1^, Xin Yu^1^, Jianni Yang^2,3^, Jun Xu^2^, Hongxia Fan^1,*^, Junxia Zheng^1,*^

^1^ *School of Biomedical and Pharmaceutical Sciences, Guangdong University of Technology, Guangzhou, 510006, PR China*

^2^ *College of Pharmacy, Jinan University, Guangzhou, 510632, PR China*

^3^ *Department of Oncology, The First Affiliated Hospital of Jinan University, Guangzhou, 510632, P. R. China*

* Corresponding authors at: School of Biomedical and Pharmaceutical Sciences, Guangdong University of Technology, China.

E-mail addresses: fanhongxia0628@126.com (Hongxia Fan), junxiazheng@gdut.edu.cn (Junxia Zheng).

**Table S1.** Primer sequences of *lasI*, *lasR*, *lasA*, *lasB*, *rhIR*, *rhII*, *pqsA* and *pqsR*

| Primer name | Sequence (5’ to 3’) | Fragment  length（bp） | Annealing  temperature（℃） |
| --- | --- | --- | --- |
| PA-*lasI*-S | ACTCAGCCGTTTCGCCATC | 155 | 60 |
| PA-*lasI*-A | TCATCATCTTCTCCACGCCTAC |  |  |
| PA-*lasR*-S | TTCATCGTCGGCAACTACCC | 296 | 60 |
| PA-*lasR*-A | GGCAGGACCGACTCTATGAAA |  |  |
| PA-*lasA*-S | ACCAGATCCAGGTGAGCAACG | 127 | 60 |
| PA-*lasA*-A | AGCGAGAAATGCAGGTGCG |  |  |
| PA-*lasB*-S | AGGAAGTCGTTCTTGCCGC | 170 | 60 |
| PA-*lasB*-A | TTCTATCCGCTGGTGTCGCT |  |  |
| PA-*rhlR*-S | CGCCATACGATTCCCTTCA | 264 | 60 |
| PA-*rhlR*-A | GGAAAGCACGCTGAGCAAAT |  |  |
| PA-*rhlI*-S | CGCAAACCCGCTACATCGT | 218 | 60 |
| PA-*rhlI*-A | TTGCAGGCTGGACCAGAATA |  |  |
| PA-*pqsA*-S | CCCTTTGCTCGACGATTTCTC | 122 | 60 |
| PA-*pqsA*-A | CGGTGGAACCCGAGGTGTAT |  |  |
| PA-*pqsR*-S | GCGACGATCAAGCAGGACA | 146 | 60 |
| PA-*pqsR*-A | GAGGCATTGCACAACGGGT |  |  |

**Table S2.** MIC values of RSE, SFA, SFE, and azithromycin

| Compound | MIC |
| --- | --- |
| RSE | >512 μg |
| SFA | >1000 μM |
| SFE | >1000 μM |
| Azithromycin | 32 μg (~ 42.7μM) |

**Table S3.** Composition analysis of Raphani Semen extract by UPLC‑Q‑Orbitrap‑MS/MS (negative ion mode)

| NO. | RT(min) | Ion | Observed (m/z) | Formula | Fragment Ions (MS/MS) | Identity | Reference |
| --- | --- | --- | --- | --- | --- | --- | --- |
| N1 | 1.05 | [M-H]- | 382.44128 | C_13_H_24_O_13_ | 382, 341, 281, 221, 179, 161, 143, 119, 101, 89, 59 | A | (Feng et al., 2020) |
| N2 | 2.5 | [M-H]- | 406.04715 | C_11_H_21_NO_9_S_3_ | 406, 363, 342, 227, 207, 195, 174, 110, 97, 75, 65 | Glucoibervirin | (Maldini et al., 2012) |
| N3 | 2.58 | [M-H]- | 295.06729 | C_15_H_16_O_8_ | 295, 235, 205, 179, 161, 143, 133, 113, 101, 89, 71, 59 | B | (Llorach et al., 2008) |
| N4 | 2.95 | [M-H]- | 438.02557 | C_11_H_21_KNO_11_S_3_ | 438, 423, 238, 204, 177, 166, 97, 75 | Glucocheirolin | (Sut et al., 2018) |
| N5 | 3.01 | [M-H]- | 436.02295 | C_12_H_23_NO_10_S_3_ | 436, 420, 372, 259, 238, 204, 178, 119, 97, 75 | Sulforaphane glucosinolate | (Mellon et al., 2002, Zabaras et al., 2013, Capriotti et al., 2018, Hooshmand et al., 2021) |
| N6 | 3.87 | [M-H]- | 437.02527 | C_15_H_20_KNO_10_S_2_ | 437, 422, 373, 519, 178, 97, 75 | Glucobarbarin | (Assefa et al., 2019) |
| N7 | 4.28 | [M-H]- | 434.02515 | C_12_H_21_NO_10_S_3_ | 434, 419, 354, 294, 259, 195, 128, 97, 75 | Glucoraphasatin sulfoxide | (Liu et al., 2020) |
| N8 | 4.36 | [M-H]- | 358.20756 | C_10_H_6_KNO_9_S_2_ | 358, 336, 195, 161, 116, 97, 75, 67 | Sinigrin | (Zabaras et al., 2013, Rakpenthai et al., 2019) |
| N9 | 4.44 | [M-H]- | 450.05704 | C_13_H_25_NO_10_S_3_ | 452, 386, 259, 192, 179, 97, 75 | Glucoalyssinin | (Zabaras et al., 2013, Bell et al., 2015, Hooshmand et al., 2021) |
| N10 | 4.69 | [M-H]- | 167.3482 | C_8_H_8_O_4_ | 167, 164, 152, 138, 126, 123, 108, 95, 69 | Vanillic acid | (Santos et al., 2014, Oniszczuk et al., 2016) |
| N11 | 5.45 | [M-H]- | 462.02081 | C_15_H_29_NO_9_S_3_ | 462, 446, 349, 311, 245, 197, 97, 62 | 7-Methylthioheptyl glucosinolate | (Hooshmand et al., 2021) |
| N12 | 5.79 | [M-H]- | 153.01917 | C_7_H_6_O_4_ | 153, 123, 109, 106, 95, 85 | 3,4-dihydroxybenzoic acid | (Vallverdu-Queralt et al., 2013, Zhong et al., 2020) |
| N13 | 6.17 | [M-H]- | 547.16797 | C_23_H_32_O_15_ | 547, 427, 341, 295, 265, 223, 205, 190, 179, | Sibiricose A1 | (Picchi et al., 2020) |
| N14 | 6.93 | [M-H]- | 163.03966 | C_9_H_8_O_3_ | 163, 135, 119, 116, 95, | p-coumaric acid | (Lin et al., 2009, Vallverdu-Queralt et al., 2013, Santos et al., 2014, Oniszczuk et al., 2016) |
| N15 | 7.91 | [M-H]- | 179.03471 | C_9_H_8_O_4_ | 179, 142, 135, 121, 97, | Caffeic acid | (Santos et al., 2014, Bell et al., 2015) |
| N16 | 7.91 | [M-H]- | 193.05029 | C_10_H_10_O_4_ | 193, 178, 149, 137, 134, 117, 85 | Ferulic acid | (Lin et al., 2009, Santos et al., 2014, Oniszczuk et al., 2016) |
| N17 | 7.98 | [M-H]- | 181.05025 | C_9_H_10_O_4_ | 181, 166, 151, 97 | Syringaldehyde | (Lin et al., 2009, Geduk et al., 2021) |
| N18 | 8.09 | [M-H]- | 163.03973 | C_9_H_8_O_3_ | 163, 148, 134, 119, 107, 95, 91, 76, 56 | 3-Coumaric acid | (Oniszczuk et al., 2016) |
| N19 | 8.18 | [M-H]- | 233.08098 | C_11_H_12_O_5_ | 223, 208, 193, 179, 164, 152, 149 | Sinapinic acid | (Geduk et al., 2021) |
| N20 | 8.5 | [M-H]- | 418.03046 | C_12_H_21_NO_9_S_3_ | 418, 399, 322, 259, 224, 176, 119, 97,75 | Glucoraphenin sulfide | (Liu et al., 2020) |
| N21 | 9.22 | [M-H]- | 137.02415 | C_7_H_6_O_3_ | 137, 112, 93 | Salicylic acid | (Oniszczuk et al., 2016) |
| N22 | 9.32 | [M-H]- | 593.15381 | C_27_H_30_O_15_ | 593, 447, 430, 285, 255, 122, 70 | Kaempferol 3-rhamnoside 7-glucoside | (Prescott et al., 2013, Vallverdu-Queralt et al., 2013, Iyda et al., 2019) |
| N23 | 10.16 | [M-H]- | 187.09735 | C_9_H_16_O_4_ | 187, 169, 125, 97 | Azelaic Acid | (Yao et al., 2022) |
| N24 | 10.3 | [M-H]- | 308.0899 | C_12_H_23_NO_6_S | 308, 267, 228, 209, 146, 97, 59 | Desulfo-2-methylbutylglucosinolate | (Huang et al., 2021) |
| N25 | 11.28 | [M-H]- | 477.10342 | C_22_H_22_O_12_ | 477, 327, 314, 285, 271, 243 | isorhamnetin 7-*O*-glucoside | (Francisco et al., 2009) |
| N26 | 11.28 | [M-H]- | 477.10342 | C_22_H_22_O_12_ | 477, 314, 285, 271, 243, 228, 123, 97, 61 | Isorhamnetin-3-*O*-glucoside | (Lin et al., 2009, Jiang et al., 2013, Bell et al., 2015) |
| N27 | 11.33 | [M-H]- | 402.0889 | C_13_H_23_NO_9_S_2_ | 402, 259, 148, 97, 75 | Pronapoleiferin | (Liu et al., 2020) |
| N28 | 11.49 | [M-H]- | 753.22559 | C_34_H_42_O_19_ | 753, 547, 460, 367, 265, 223, 205, 164 | 1,2-disinapoylgentiobiose | (Vallejo et al., 2003, Zabaras et al., 2013, Villarreal-Garcia et al., 2016, An et al., 2021) |
| N29 | 11.65 | [M-H]- | 609.17883 | C_27_H_30_O_16_ | 609, 301 | Rutin | (Gratacos-Cubarsi et al., 2010, Santos et al., 2014) |
| N30 | 11.85 | [M-H]- | 723.20923 | C_33_H_40_O_18_ | 723, 677, 517, 265, 208, 175, 160, 130 | 2-Feruloyl-1-sinapoylgentiobiose | (Vallejo et al., 2003, Villarreal-Garcia et al., 2016, Picchi et al., 2020) |
| N31 | 15.18 | [M-H]- | 959.28125 | C_34_H_42_O_19_ | 959, 735, 529,367, 223, 205, 190, 164 | 1,2,2'-Trisinapoylgentiobiose | (Vallejo et al., 2003, Lin et al., 2009, Villarreal-Garcia et al., 2016) |
| N32 | 15.37 | [M-H]- | 431.09814 | C_21_H_20_O_10_ | 431, 344, 285, 257, 151, 107 | Kaempferol 3-*O*-α-L-rhamnopyranoside | (Ibrahim et al., 2016) |
| N33 | 17.16 | [M-H]- | 591.1037 | C_18_H_32_H_14_ | 591, 527, 429, 414, 364, 235, 223, 205, 190, 184, 130, 97 | 1,2-Disinapoylglucose | (Lin et al., 2009, Jiang et al., 2013) |
| N34 | 17.27 | [M-H]- | 285.04044 | C_15_H_10_O_16_ | 285, 221, 174, 87 | Kaempferol | (Santos et al., 2014) |
| N35 | 18.12 | [M-H]- | 182.92944 | C_8_H_8_O_5_ | 183, 139, 123, 97, 85, 61 | 3,5-dihydroxy-4-methoxybenzoic acid | (Zhong et al., 2020) |
| N36 | 20.59 | [M-H]- | 191.10751 | C_10_H_9_NO_3_ | 191, 155, 144, 103, 79, 61 | 2-(5-hydroxy-1H-indol-3-yl)acetic acid | (Grant et al., 2021) |
| N37 | 21.99 | [M-H]- | 300.99875 | C_14_H_6_O_8_ | 301, 291, 254, 233, 214, 168, 126 | Ellagic acid | (Sepúlveda et al., 2018) |
| N38 | 23.94 | [M-H]- | 299.20154 | C_16_H_12_O_6_ | 299, 180, 132 | Diosmetin | (Santos et al., 2014) |
| N39 | 26.22 | [M-H]- | 255.23288 | C_16_H_32_O_2_ | 255.23288 | Palmitic Acid | (Li et al., 2020) |
| N40 | 26.57 | [M-H]- | 281.24838 | C_18_H_34_O_2_ | 281.24838 | Oleic acid | (Li et al., 2020) |
| N41 | 27.14 | [M-H]- | 409.26282 | C_14_H_19_NO_9_S_2_ | 409, 373, 345, 189, 145, 121, 97, 79 | Glucotropaeolin | (Li et al., 2020) |
| N42 | 27.39 | [M-H]- | 283.26401 | C_18_H_36_O_2_ | 283.26401 | Stearic acid | (Li et al., 2020) |

*A = 2,3,4,5,6-pentahydroxy-7-[(2S,3R,4S,5S,6R)-3,4,5-trihydroxy-6-(hydroxymethyl)oxan-2-yl]oxyheptanoic acid, B = 2-[3-(3,4-dimethoxyphenyl)prop-2-enoyloxy]butanedioic acid.

**Table S4.** Composition analysis of Raphani Semen extract UPLC-Q-Orbitrap-MS/MS (positive ion mode)

| NO. | RT(min) | Ion | Observed (m/z) | Formula | Fragment Ions (MS/MS) | Identity | Reference |
| --- | --- | --- | --- | --- | --- | --- | --- |
| P1 | 0.99 | [M+H]+ | 134.11752 | C_4_H_7_NO_4_ | 134, 117, 105, 87, 70, 63 | Aspartic acid | (Zhong et al., 2020) |
| P2 | 1.06 | [M+H]+ | 147.02217 | C_5_H_9_NS_2_ | 147, 101, | Iberverin | (Agerbirk et al., 2015) |
| P3 | 1.15 | [M+H]+ | 209.12811 | C_11_H_17_N_2_O_2_+ | 209, 204, 150, 106, 91, 78, 61 | A | (Boettcher et al., 2009) |
| P4 | 1.38 | [M+H]+ | 146.96121 | C_6_H_14_N_2_O_2_ | 147, 128, 123, 119, 101, 88, 83, 71, 65, 55 | Lysine | (Zhong et al., 2020) |
| P5 | 1.54 | [M+H]+ | 132.10188 | C_6_H_13_NO_2_ | 132, 114, 97, 91, 86, 72, 68, 61 | Leucine | (Zhong et al., 2020) |
| P6 | 1.69 | [M+H]+ | 416.19119 | C_19_H_30_NO_9_+ | 416, 357, 254, 195, 151, 101, 76 | B | (Boettcher et al., 2009) |
| P7 | 2.13 | [M+H]+ | 166.02966 | C_9_H_11_NO_2_ | 166, 131, 120, 103, 88,55 | Phenylalanine | (Zhong et al., 2020) |
| P8 | 2.56 | [M+H]+ | 195.08746 | C_8_H_10_N_4_O_2_ | 195, 176, 163, 138, 133, 107, 96, 89, 71, 55 | Caffeine | (Zhong et al., 2020) |
| P9 | 3.42 | [M+H]+ | 254.13817 | C_13_H_20_NO_4_+ | 254, 195, 180, 151, 162, 151, 126, 109, 73 | C | (Boettcher et al., 2009) |
| P10 | 3.92 | [M+H]+ | 266.1384 | C_14_H_20_NO_4_+ | 266, 207, 163, 145, 128, 89, 57 | D | (Boettcher et al., 2009) |
| P11 | 4.06 | [M+H]+ | 472.21756 | C_22_H_34_NO_10_+ | 472, 310, 251, 207, 175, 91 | E | (Boettcher et al., 2009) |
| P12 | 4.37 | [M+H]+ | 296.1489 | C_15_H_22_NO_5_+ | 296, 237, 193, 178, 161, 133, 105, 86, 63 | F | (Boettcher et al., 2009) |
| P13 | 4.56 | [M+H]+ | 124.03934 | C_6_H_5_NO_2_ | 124, 96, 84, 80, | nicotinic acid | (Yao et al., 2022) |
| P14 | 4.84 | [M+H]+ | 192.10139 | C_7_H_13_NOS_2_ | 192, 168, 146, 128, 123, 110, 90, 87,80, 61 | 1-isothiocyanato-5-methylsulfinylpentane | (Song et al., 2005) |
| P15 | 4.99 | [M+H]+ | 208.13304 | C_12_H_18_NO_2_+ | 208, 177, 149, 143, 125, 105, 84, 63 | Benzoylcholine | (Boettcher et al., 2009) |
| P16 | 5.41 | [M+H]+ | 638.28027 | C_31_H_44_NO_13_+ | 638, 579, 462, 280, 237, 221, 177, 108 | G | (Boettcher et al., 2009) |
| P17 | 5.62 | [M+H]+ | 178.03543 | C_6_H_11_NOS_2_ | 178, 161, 147, 135, 130, 118, 113, 98, 88, 81, 55 | Sulforaphane | (Song et al., 2005) |
| P18 | 5.76 | [M+H]+ | 450.2128 | C_23_H_32_NO_8_ | 450, 409, 391, 253, 226, 195, 151, 109 | H | (Boettcher et al., 2009) |
| P19 | 5.95 | [M+H]+ | 280.15396 | C_15_H_22_NO_4_+ | 280, 258, 221, 177, 145, 117, 89, 61 | 4-Hydroxy-3-methoxycinnamoylcholine | (Song et al., 2005) |
| P20 | 6.24 | [M+H]+ | 310.16437 | C_16_H_24_NO_5_+ | 310, 251, 236, 207, 175, 147, 119, 91, 65 | Sinapoylcholine | (Boettcher et al., 2009) |
| P21 | 7.37 | [M+H]+ | 357.20972 | C_16_H_20_O_9_ | 357, 321, 212, 184, 177, 145, | Gentiopicroside | (Nzekoue et al., 2020) |
| P22 | 7.95 | [M+H]+ | 620.26959 | C_31_H_42_NO_12_+ | 620, 561, 543, 399, 381, 369, 339, 291, 242, 151, 83 | I | (Boettcher et al., 2009) |
| P23 | 8.25 | [M+H]+ | 176.0197 | C_6_H_9_NOS_2_ | 176, 117, 125, 112, 103, 87, 85, 78, 65, 53 | Sulforaphene | (Agerbirk et al., 2015) |
| P24 | 8.42 | [M+H]+ | 294.17047 | C_16_H_24_NO_4_+ | 294, 235, 191, 174, 155, 128, 94, 61 | 3,4-Dimethoxycinnamoylcholine | (Boettcher et al., 2009) |
| P25 | 9.93 | [M+H]+ | 488.22739 | C_26_H_34_NO_8_+ | 488, 429, 414, 357, 325, 275, 231, 181, 146, 99 | J | (Boettcher et al., 2009) |
| P26 | 10.46 | [M+H]+ | 458.21658 | C_25_H_32_NO_7_+ | 458, 399, 381, 369, 349, 309, 277, 245, 162, 137, 94 | K | (Boettcher et al., 2009) |
| P27 | 11.4 | [M+H]+ | 164.10706 | C_5_H_9_NOS_2_ | 164, 146, 141, 118, 107, 100, 95, 82, 65, 53 | Iberin | (Song et al., 2005) |
| P28 | 23.7 | [M+H]+ | 156.13843 | C_6_H_9_N_3_O_2_ | 156, 139, 121, 114, 110, 100, 97, 93, 86, 81, 71, 69, 67, 57, 55 | Histidine | (Zhong et al., 2020) |
| P29 | 26.55 | [M+H]+ | 118.58999 | C_4_H_5_NS | 118, 100, 83, 72, 59, 58, 55 | Allyl isothiocyanate | (Song et al., 2005) |

*A=*N*,*N*,*N*-Trimethyl-2-[(3-pyridinylcarbonyl)oxy]ethanaminium; B=2-[[4-(Hexopyranosyloxy)-3-methoxybenzoyl]oxy]-*N*,*N*,*N*-trimethylethanaminium; C=2-[(4-Hydroxy-3-methoxybenzoyl)oxy]-*N*,*N*,*N*-trimethylethanaminium-4-Hydroxy-3-methoxybenzoylcholine; D=2-[3-(3,4-dihydroxyphenyl)prop-2-enoyloxy]ethyl-trimethylazanium; E=2-[[3-[4-(Hexopyranosyloxy)-3,5-dimethoxyphenyl]-1-oxo-2-propen-1-yl]oxy]-*N*,*N*,*N*-trimethylethanaminium; F=2-[[3-(3,4-Dihydroxy-5-methoxyphenyl)-1-oxo-2-propen-1-yl]oxy]-*N*,*N*,*N*-trimethylethanaminium; G=2-[[3-[4-[2-[4-(Hexopyranosyloxy)-3-methoxyphenyl]-2-hydroxy-1-(hydroxymethyl)ethoxy]-3-methoxyphenyl]-1-oxo-2-propen-1-yl]oxy]-*N*,*N*,*N*-trimethylethanaminium; H=2-[[4-[2-Hydroxy-2-(4-hydroxy-3-methoxyphenyl)-1-(hydroxymethyl)ethoxy]-3-methoxybenzoyl]oxy]-*N*,*N*,*N*-trimethylethanaminium; I=2-[[3-[2-[4-(Hexopyranosyloxy)-3-methoxyphenyl]-2,3-dihydro-3-(hydroxymethyl)-7-methoxy-5-benzofuranyl]-1-oxo-2-propen-1-yl]oxy]-*N*,*N*,*N*-trimethylethanaminium; J=2-[[4-[2-Hydroxy-2-(4-hydroxy-3-methoxyphenyl)-1-(hydroxymethyl)ethoxy]-3-methoxybenzoyl]oxy]-*N*,*N*,*N*-trimethylethanaminium; K=2-[[3-[2,3-Dihydro-2-(4-hydroxy-3-methoxyphenyl)-3-(hydroxymethyl)-7-methoxy-5-benzofuranyl]-1-oxo-2-propen-1-yl]oxy]-*N*,*N*,*N*-trimethylethanaminium.

**Fig. S1** Analysis and summary of chemical composition of RSE. The figure shows the classification of the 71 compounds identified by UPLC‑Q‑Orbitrap‑MS/MS, including glucosinolates (12), isothiocyanates (6), phenolic acids (15), sinapic acid esters (15), flavonoids (7), and other metabolites (16).

**Fig. S2** Inhibition of biofilm formation by SFA and SFE. (A, B) Biofilm biomass measured by crystal violet staining after treatment with increasing concentrations of SFA (A) or SFE (B). AZM: azithromycin (positive control, 32 μg/mL). (C, D) COMSTAT analysis of total biofilm biomass for SFA (C) and SFE (D). (E, F) COMSTAT analysis of average biofilm thickness for SFA (E) and SFE (F). (G, H) Representative CLSM images of 24 h biofilms treated with SFA (G) or SFE (H). All data are presented as mean ± SD from three independent biological replicates (n = 3). Statistical significance was determined by one‑way ANOVA followed by Tukey’s post‑hoc test. **P* < 0.05, ***P* < 0.01, ****P* < 0.001, *****P* < 0.0001 compared to the control.

**Fig. S3** Effects of SFA and SFE on motility and virulence factors. (A–D) Swimming and swarming motility zones after treatment with SFA (A, B) or SFE (C, D). (E–G) Pyocyanin (E), rhamnolipid (F) and extracellular polysaccharide (G) production after SFA treatment. (H–J) Pyocyanin (H), rhamnolipid (I) and extracellular polysaccharide (J) production after SFE treatment. AZM: azithromycin (positive control, 32 μg/mL). Data are presented as mean ± SD from three independent biological replicates (n = 3). One‑way ANOVA with Tukey’s post‑hoc test was used. **P* < 0.05, ***P* < 0.01, ****P* < 0.001, *****P* < 0.0001 compared to the control.

**Fig. S4** Agarose gel electrophoresis of PCR products. PCR products for the target genes (16S rRNA, lasI, lasR, lasA, lasB, rhlI, rhlR, pqsR, pqsA) were separated on a 1.5% agarose gel. Lane M: DNA marker (e.g., 100 bp ladder). The expected amplicon sizes are listed in Table 1.

**Fig. S5** Amplification curves (primary curves) of qRT‑PCR. (A) *16S rRNA,* (B) *lasI*, (C) *lasR*, (D) *lasA*, (E) *lasB*, (F) *rhlI*, (G) *rhlR*, (H) *pqsR*, (I) *pqsA*. Fluorescence was plotted against cycle number for each gene.

**Fig.S6** Melting curves of qRT‑PCR. (A) *16S rRNA,* (B) *lasI*, (C) *lasR*, (D) *lasA*, (E) *lasB*, (F) *rhlI*, (G) *rhlR*, (H) *pqsR*, (I) *pqsA*. The negative first derivative of fluorescence (−dF/dT) was plotted against temperature to verify the specificity of each amplicon.

**Fig.S7** The MS/MS Spectrum of N1

**Fig.S8** The MS/MS Spectrum of N2

**Fig.S9** The MS/MS Spectrum of N3

**Fig.S10** The MS/MS Spectrum of N4

**Fig.S11** The MS/MS Spectrum of N5

**Fig.S12** The MS/MS Spectrum of N6

**Fig.S13** The MS/MS Spectrum of N7

**Fig.S14** The MS/MS Spectrum of N8

**Fig.S15** The MS/MS Spectrum of N9

**Fig.S16** The MS/MS Spectrum of N10

**Fig.S17** The MS/MS Spectrum of N11

**Fig.S18** The MS/MS Spectrum of N12

**Fig.S19** The MS/MS Spectrum of N13

**Fig.S20** The MS/MS Spectrum of N14

**Fig.S21** The MS/MS Spectrum of N15

**Fig.S22** The MS/MS Spectrum of N16

**Fig.S23** The MS/MS Spectrum of N17

**Fig.S24** The MS/MS Spectrum of N18

**Fig.S25** The MS/MS Spectrum of N19

**Fig.S26** The MS/MS Spectrum of N20

**Fig.S27** The MS/MS Spectrum of N21

**Fig.S28** The MS/MS Spectrum of N22

**Fig.S29** The MS/MS Spectrum of N23

**Fig.S30** The MS/MS Spectrum of N24

**Fig.S31** The MS/MS Spectrum of N25

**Fig.S32** The MS/MS Spectrum of N26

**Fig.S33** The MS/MS Spectrum of N27

**Fig.S34** The MS/MS Spectrum of N28

**Fig.S35** The MS/MS Spectrum of N29

**Fig.S36** The MS/MS Spectrum of N30

**Fig.S37** The MS/MS Spectrum of N31

**Fig.S38** The MS/MS Spectrum of N32

**Fig.S39** The MS/MS Spectrum of N33

**Fig.S40** The MS/MS Spectrum of N34

**Fig.S41** The MS/MS Spectrum of N35

**Fig.S42** The MS/MS Spectrum of N36

**Fig.S43** The MS/MS Spectrum of N37

**Fig.S44** The MS/MS Spectrum of N38

**Fig.S45** The MS/MS Spectrum of N39

**Fig.S46** The MS/MS Spectrum of N40

**Fig.S47** The MS/MS Spectrum of N41

**Fig.S48** The MS/MS Spectrum of N42

**Fig.S49** The MS/MS Spectrum of P1

**Fig.S50** The MS/MS Spectrum of P2

**Fig.S51** The MS/MS Spectrum of P3

**Fig.S52** The MS/MS Spectrum of P4

**Fig.S53** The MS/MS Spectrum of P5

**Fig.S54** The MS/MS Spectrum of P6

**Fig.S55** The MS/MS Spectrum of P7

**Fig.S56** The MS/MS Spectrum of P8

**Fig.S57** The MS/MS Spectrum of P9

**Fig.S58** The MS/MS Spectrum of P10

**Fig.S59** The MS/MS Spectrum of P11

**Fig.S60** The MS/MS Spectrum of P12

**Fig.S61** The MS/MS Spectrum of P13

**Fig.S62** The MS/MS Spectrum of P14

**Fig.S63** The MS/MS Spectrum of P15

**Fig.S64** The MS/MS Spectrum of P16

**Fig.S65** The MS/MS Spectrum of P17

**Fig.S66** The MS/MS Spectrum of P18

**Fig.S67** The MS/MS Spectrum of P19

**Fig.S68** The MS/MS Spectrum of P20

**Fig.S69** The MS/MS Spectrum of P21

**Fig.S70** The MS/MS Spectrum of P22

**Fig.S71** The MS/MS Spectrum of P23

**Fig.S72** The MS/MS Spectrum of P24

**Fig.S73** The MS/MS Spectrum of P25

**Fig.S74** The MS/MS Spectrum of P26

**Fig.S75** The MS/MS Spectrum of P27

**Fig.S76** The MS/MS Spectrum of P28

**Fig.S77** The MS/MS Spectrum of P29

References

Agerbirk, N. and C. E. Olsen, 2015. Glucosinolate hydrolysis products in the crucifer Barbarea vulgaris include a thiazolidine-2-one from a specific phenolic isomer as well as oxazolidine-2-thiones. PHYTOCHEMISTRY. 115, 143-151. <https://doi.org/10.1016/j.phytochem.2014.11.002>

An, H.-M., M.-N. Li, H. Yang, et al., 2021. A validated UHPLC-MS/MS method for pharmacokinetic and brain distribution studies of twenty constituents in rat after oral administration of Jia-Wei-Qi-Fu-Yin. JOURNAL OF PHARMACEUTICAL AND BIOMEDICAL ANALYSIS. 202, <https://doi.org/10.1016/j.jpba.2021.114140>

Assefa, A. D., S. Choi, J. E. Lee, et al., 2019. Determination of glucosinolate contents in Brassica germplasm collections and inter- & intra-leaves distribution pattern using UPLC-MS/MS Multiple Reaction Monitoring scan mode. bioRxiv.

Bell, L., M. J. Oruna-Concha and C. Wagstaff, 2015. Identification and quantification of glucosinolate and flavonol compounds in rocket salad (Eruca sativa, Eruca vesicaria and Diplotaxis tenuifolia) by LC-MS: Highlighting the potential for improving nutritional value of rocket crops. Food Chem. 172, 852-861. <https://doi.org/10.1016/j.foodchem.2014.09.116>

Boettcher, C., E. von Roepenack-Lahaye, J. Schmidt, et al., 2009. Analysis of phenolic choline esters from seeds of Arabidopsis thaliana and Brassica napus by capillary liquid chromatography/electrospray-tandem mass spectrometry. JOURNAL OF MASS SPECTROMETRY. 44, 466-476. <https://doi.org/10.1002/jms.1522>

Capriotti, A. L., C. Cavaliere, G. La Barbera, et al., 2018. Chromatographic column evaluation for the untargeted profiling of glucosinolates in cauliflower by means of ultra-high performance liquid chromatography coupled to high resolution mass spectrometry. TALANTA. 179, 792-802. <https://doi.org/10.1016/j.talanta.2017.12.019>

Feng, J., P. Yu, Q. Zhou, et al., 2020. An integrated data filtering and identification strategy for rapid profiling of chemical constituents, with Arnebiae Radix as an example. JOURNAL OF CHROMATOGRAPHY A. 1629, <https://doi.org/10.1016/j.chroma.2020.461496>

Francisco, M., D. A. Moreno, M. Elena Cartea, et al., 2009. Simultaneous identification of glucosinolates and phenolic compounds in a representative collection of vegetable Brassica rapa. JOURNAL OF CHROMATOGRAPHY A. 1216, 6611-6619. <https://doi.org/10.1016/j.chroma.2009.07.055>

Geduk, A. S. and F. Zengin, 2021. LC-MS/MS characterization, antidiabetic, antioxidative, and antibacterial effects of different solvent extracts of Anamur banana (Musa Cavendishii). FOOD SCIENCE AND BIOTECHNOLOGY. 30, 1183-1193. <https://doi.org/10.1007/s10068-021-00953-5>

Grant, K. M., C. Livie, K. Smith, et al., 2021. A rapid liquid chromatography-tandem mass spectrometry method for the analysis of urinary 5-hydroxyindoleacetic acid (5-HIAA) that incorporates a <SUP>13</SUP>C-labelled internal standard. ANNALS OF CLINICAL BIOCHEMISTRY. 58, 622-631. <https://doi.org/10.1177/00045632211038021>

Gratacos-Cubarsi, M., A. Ribas-Agusti, J. A. Garcia-Regueiro, et al., 2010. Simultaneous evaluation of intact glucosinolates and phenolic compounds by UPLC-DAD-MS/MS in Brassica oleracea L. var. botrytis. Food Chem. 121, 257-263. <https://doi.org/10.1016/j.foodchem.2009.11.081>

Hooshmand, K. and I. S. Fomsgaard, 2021. Analytical Methods for Quantification and Identification of Intact Glucosinolates in Arabidopsis Roots Using LC-QqQ(LIT)-MS/MS. METABOLITES. 11, <https://doi.org/10.3390/metabo11010047>

Huang, Q., F. Zhang, S. Liu, et al., 2021. Systematic investigation of the pharmacological mechanism for renal protection by the leaves of Eucommia ulmoides Oliver using UPLC-Q-TOF/MS combined with network pharmacology analysis. BIOMEDICINE & PHARMACOTHERAPY. 140, <https://doi.org/10.1016/j.biopha.2021.111735>

Ibrahim, L., A. Elkhateeb, M. Marzouk, et al., 2016. Flavonoid investigation, LC–ESI-MS profile and cytotoxic activity of Raphanus raphanistrum L. (Brassicaceae). Journal of Chemical and Pharmaceutical Research.

Iyda, J. H., A. Fernandes, F. D. Ferreira, et al., 2019. Chemical composition and bioactive properties of the wild edible plant Raphanus raphanistrum L. FOOD RESEARCH INTERNATIONAL. 121, 714-722. <https://doi.org/10.1016/j.foodres.2018.12.046>

Jiang, J., Y. Shao, A. Li, et al., 2013. Phenolic Composition Analysis and Gene Expression in Developing Seeds of Yellow- and Black-seeded Brassica napus. JOURNAL OF INTEGRATIVE PLANT BIOLOGY. 55, 537-551. <https://doi.org/10.1111/jipb.12039>

Li, X., M. Sdiri, J. Peng, et al., 2020. Identification and characterization of chemical components in the bioactive fractions of Cynomorium coccineum that possess anticancer activity. INTERNATIONAL JOURNAL OF BIOLOGICAL SCIENCES. 16, 61-73. <https://doi.org/10.7150/ijbs.38475>

Lin, L.-Z. and J. M. Harnly, 2009. Identification of the Phenolic Components of Collard Greens, Kale, and Chinese Broccoli. JOURNAL OF AGRICULTURAL AND FOOD CHEMISTRY. 57, 7401-7408. <https://doi.org/10.1021/jf901121v>

Liu, Y., M. Rossi, X. Liang, et al., 2020. An Integrated Metabolomics Study of Glucosinolate Metabolism in Different Brassicaceae Genera. METABOLITES. 10, <https://doi.org/10.3390/metabo10080313>

Llorach, R., A. Martinez-Sanchez, F. A. Tomas-Barberan, et al., 2008. Characterisation of polyphenols and antioxidant properties of five lettuce varieties and escarole. Food Chem. 108, 1028-1038. <https://doi.org/10.1016/j.foodchem.2007.11.032>

Maldini, M., S. Baima, G. Morelli, et al., 2012. A liquid chromatography-mass spectrometry approach to study "glucosinoloma" in broccoli sprouts. JOURNAL OF MASS SPECTROMETRY. 47, 1198-1206. <https://doi.org/10.1002/jms.3028>

Mellon, F. A., R. N. Bennett, B. Holst, et al., 2002. Intact glucosinolate analysis in plant extracts by programmed cone voltage electrospray LC/MS: Performance and comparison with LC/MS/MS methods. ANALYTICAL BIOCHEMISTRY. 306, 83-91. <https://doi.org/10.1006/abio.2002.5677>

Nzekoue, F. K., S. Angeloni, L. Navarini, et al., 2020. Coffee silverskin extracts: Quantification of 30 bioactive compounds by a new HPLC-MS/MS method and evaluation of their antioxidant and antibacterial activities. FOOD RESEARCH INTERNATIONAL. 133, <https://doi.org/10.1016/j.foodres.2020.109128>

Oniszczuk, A. and M. Olech, 2016. Optimization of ultrasound-assisted extraction and LC-ESI-MS/MS analysis of phenolic acids from Brassica oleracea L. var. sabellica. INDUSTRIAL CROPS AND PRODUCTS. 83, 359-363. <https://doi.org/10.1016/j.indcrop.2016.01.015>

Picchi, V., R. Lo Scalzo, A. Tava, et al., 2020. Phytochemical Characterization and In Vitro Antioxidant Properties of Four Brassica Wild Species from Italy. MOLECULES. 25, <https://doi.org/10.3390/molecules25153495>

Prescott, T. A. K., G. C. Kite, E. A. Porter, et al., 2013. Highly glycosylated flavonols with an O-linked branched pentasaccharide from Iberis saxatilis (Brassicaceae). PHYTOCHEMISTRY. 88, 85-91. <https://doi.org/10.1016/j.phytochem.2012.12.009>

Rakpenthai, A., G. Khaksar, M. Burow, et al., 2019. Metabolic Changes and Increased Levels of Bioactive Compounds in White Radish (Raphanus sativus L. cv. 01) Sprouts Elicited by Oligochitosan. AGRONOMY-BASEL. 9, <https://doi.org/10.3390/agronomy9080467>

Santos, J., M. B. P. P. Oliveira, E. Ibanez, et al., 2014. Phenolic profile evolution of different ready-to-eat baby-leaf vegetables during storage. JOURNAL OF CHROMATOGRAPHY A. 1327, 118-131. <https://doi.org/10.1016/j.chroma.2013.12.085>

Sepúlveda, L., J. E. Wong-Paz, J. Buenrostro-Figueroa, et al., 2018. Solid state fermentation of pomegranate husk: Recovery of ellagic acid by SEC and identification of ellagitannins by HPLC/ESI/MS. Food Bioscience. <https://doi.org/10.1016/j.fbio.2018.01.006>

Song, L. J., J. J. Morrison, N. P. Botting, et al., 2005. Analysis of glucosinolates, isothiocyanates, and amine degradation products in vegetable extracts and blood plasma by LC-MS/MS. ANALYTICAL BIOCHEMISTRY. 347, 234-243. <https://doi.org/10.1016/j.ab.2005.09.040>

Sut, S., I. Boschiero, M. Solana, et al., 2018. Supercritical CO2 Extraction of Eruca sativa Using Cosolvents: Phytochemical Composition by LC-MS Analysis. MOLECULES. 23, <https://doi.org/10.3390/molecules23123240>

Vallejo, F., F. Tomás-Barberan and C. García-Viguera, 2003. Health-promoting compounds in broccoli as influenced by refrigerated transport and retail sale period. JOURNAL OF AGRICULTURAL AND FOOD CHEMISTRY. 51, 3029-3034. <https://doi.org/10.1021/jf021065j>

Vallverdu-Queralt, A., J. F. Rinaldi de Alvarenga, R. Estruch, et al., 2013. Bioactive compounds present in the Mediterranean sofrito. Food Chem. 141, 3365-3372. <https://doi.org/10.1016/j.foodchem.2013.06.032>

Villarreal-Garcia, D., V. Nair, L. Cisneros-Zevallos, et al., 2016. Plants as Biofactories: Postharvest Stress-Induced Accumulation of Phenolic Compounds and Glucosinolates in Broccoli Subjected to Wounding Stress and Exogenous Phytohormones. FRONTIERS IN PLANT SCIENCE. 7, <https://doi.org/10.3389/fpls.2016.00045>

Yao, Y., Y.-C. Yu, M.-R. Cai, et al., 2022. UPLC-MS/MS method for the determination of the herb composition of Tangshen formula and the in vivo pharmacokinetics of its metabolites in rat plasma. PHYTOCHEMICAL ANALYSIS. 33, 402-426. <https://doi.org/10.1002/pca.3098>

Zabaras, D., M. Roohani, R. Krishnamurthy, et al., 2013. Characterisation of taste-active extracts from raw Brassica oleracea vegetables. FOOD & FUNCTION. 4, 592-601. <https://doi.org/10.1039/c2fo30192j>

Zhong, J., N. Chen, S. Huang, et al., 2020. Chemical profiling and discrimination of green tea and Pu-erh raw tea based on UPLC-Q-Orbitrap-MS/MS and chemometrics. Food Chem. 326, <https://doi.org/10.1016/j.foodchem.2020.126760>
