# Supplementary material for: Raphani Semen Extract Targets the las Quorum Sensing System to Suppress Pseudomonas aeruginosa Biofilm and Signal Molecule Production
Source: Food Sci Nutr. 2026 Aug 12;14(8):e72034. doi: 10.1002/fsn3.72034 (PMC13469746; doi:10.1002/fsn3.72034)
Supplement: Supplementary file 2 [file FSN3-14-e72034-s002.docx]

**Supplementary material_2**

**Raphani Semen Extract Targets the *las* Quorum Sensing System to Suppress *Pseudomonas aeruginosa* Biofilm and Signal Molecule Production**

Juntao Xie^1^, Yujing Huang^1,2^, Honggeng Chen^1^, Zhiyun Xia^1^, Likang Wang^1^, Xin Yu^1^, Jianni Yang^2,3^, Jun Xu^2^, Hongxia Fan^1,*^, Junxia Zheng^1,*^

^1^ *School of Biomedical and Pharmaceutical Sciences, Guangdong University of Technology, Guangzhou, 510006, PR China*

^2^ *College of Pharmacy, Jinan University, Guangzhou, 510632, PR China*

^3^ *Department of Oncology, The First Affiliated Hospital of Jinan University, Guangzhou, 510632, P. R. China*

* Corresponding authors at: School of Biomedical and Pharmaceutical Sciences, Guangdong University of Technology, China.

E-mail addresses: fanhongxia0628@126.com (Hongxia Fan), junxiazheng@gdut.edu.cn (Junxia Zheng).

**Fig. S7-77 MS/MS spectra of the 71 identified compounds**

**Fig.S7** The MS/MS Spectrum of N1

**Fig.S8** The MS/MS Spectrum of N2

**Fig.S9** The MS/MS Spectrum of N3

**Fig.S10** The MS/MS Spectrum of N4

**Fig.S11** The MS/MS Spectrum of N5

**Fig.S12** The MS/MS Spectrum of N6

**Fig.S13** The MS/MS Spectrum of N7

**Fig.S14** The MS/MS Spectrum of N8

**Fig.S15** The MS/MS Spectrum of N9

**Fig.S16** The MS/MS Spectrum of N10

**Fig.S17** The MS/MS Spectrum of N11

**Fig.S18** The MS/MS Spectrum of N12

**Fig.S19** The MS/MS Spectrum of N13

**Fig.S20** The MS/MS Spectrum of N14

**Fig.S21** The MS/MS Spectrum of N15

**Fig.S22** The MS/MS Spectrum of N16

**Fig.S23** The MS/MS Spectrum of N17

**Fig.S24** The MS/MS Spectrum of N18

**Fig.S25** The MS/MS Spectrum of N19

**Fig.S26** The MS/MS Spectrum of N20

**Fig.S27** The MS/MS Spectrum of N21

**Fig.S28** The MS/MS Spectrum of N22

**Fig.S29** The MS/MS Spectrum of N23

**Fig.S30** The MS/MS Spectrum of N24

**Fig.S31** The MS/MS Spectrum of N25

**Fig.S32** The MS/MS Spectrum of N26

**Fig.S33** The MS/MS Spectrum of N27

**Fig.S34** The MS/MS Spectrum of N28

**Fig.S35** The MS/MS Spectrum of N29

**Fig.S36** The MS/MS Spectrum of N30

**Fig.S37** The MS/MS Spectrum of N31

**Fig.S38** The MS/MS Spectrum of N32

**Fig.S39** The MS/MS Spectrum of N33

**Fig.S40** The MS/MS Spectrum of N34

**Fig.S41** The MS/MS Spectrum of N35

**Fig.S42** The MS/MS Spectrum of N36

**Fig.S43** The MS/MS Spectrum of N37

**Fig.S44** The MS/MS Spectrum of N38

**Fig.S45** The MS/MS Spectrum of N39

**Fig.S46** The MS/MS Spectrum of N40

**Fig.S47** The MS/MS Spectrum of N41

**Fig.S48** The MS/MS Spectrum of N42

**Fig.S49** The MS/MS Spectrum of P1

**Fig.S50** The MS/MS Spectrum of P2

**Fig.S51** The MS/MS Spectrum of P3

**Fig.S52** The MS/MS Spectrum of P4

**Fig.S53** The MS/MS Spectrum of P5

**Fig.S54** The MS/MS Spectrum of P6

**Fig.S55** The MS/MS Spectrum of P7

**Fig.S56** The MS/MS Spectrum of P8

**Fig.S57** The MS/MS Spectrum of P9

**Fig.S58** The MS/MS Spectrum of P10

**Fig.S59** The MS/MS Spectrum of P11

**Fig.S60** The MS/MS Spectrum of P12

**Fig.S61** The MS/MS Spectrum of P13

**Fig.S62** The MS/MS Spectrum of P14

**Fig.S63** The MS/MS Spectrum of P15

**Fig.S64** The MS/MS Spectrum of P16

**Fig.S65** The MS/MS Spectrum of P17

**Fig.S66** The MS/MS Spectrum of P18

**Fig.S67** The MS/MS Spectrum of P19

**Fig.S68** The MS/MS Spectrum of P20

**Fig.S69** The MS/MS Spectrum of P21

**Fig.S70** The MS/MS Spectrum of P22

**Fig.S71** The MS/MS Spectrum of P23

**Fig.S72** The MS/MS Spectrum of P24

**Fig.S73** The MS/MS Spectrum of P25

**Fig.S74** The MS/MS Spectrum of P26

**Fig.S75** The MS/MS Spectrum of P27

**Fig.S76** The MS/MS Spectrum of P28

**Fig.S77** The MS/MS Spectrum of P29
